# Obstetric and perinatal outcomes in pregnancies conceived with donor versus partner sperm: a systematic review and meta-analysis

**DOI:** 10.3389/fendo.2025.1590261

**Published:** 2025-07-23

**Authors:** Junjie Liu, Yanpeng Dai, Zuozhe Song, Xintao Sun, Dongdong Lv, Dehua Zhao

**Affiliations:** ^1^ Henan Human Sperm Bank, The Third Affiliated Hospital of Zhengzhou University, Zhengzhou, China; ^2^ Department of Clinical Laboratory, The Third Affiliated Hospital of Zhengzhou University, Zhengzhou, China

**Keywords:** meta-analysis, donor conception, semen preservation, reproductive techniques, pregnancy outcome

## Abstract

**Background:**

Male-related factors contribute to 30-40% of infertility cases, with donor sperm serving as a critical solution for severe male infertility or paternally inherited genetic disorders. While cryopreservation ensures virological safety, concerns persist regarding sperm DNA damage, oxidative stress, and epigenetic impacts on embryogenesis. Previous studies have shown inconsistent evidence regarding obstetric and perinatal outcomes using donor versus partner sperm. This meta-analysis aimed to compare these outcomes to guide evidence-based clinical decisions.

**Methods:**

To identify studies published up to December 2024, we systematically search Embase, PubMed, Scopus, Wanfang, Web of Science, and China National Knowledge Infrastructure (CNKI). Studies investigating obstetric and perinatal outcomes using donor versus partner sperm were included regardless of the conception method. Adjusted estimates were prioritized, but crude estimates were utilized when necessary. Given the clinical and methodological heterogeneity, random-effects models were utilized to pool relative risks (RRs) and their 95% confidence intervals (CIs).

**Results:**

This analysis included 64 studies. Donor sperm was linked to better clinical pregnancy rates (RR 1.27, 95% CI 1.08–1.48) and decreased incidences of biochemical pregnancy (RR 0.85, 95% CI 0.81–0.88), miscarriage (RR 0.91, 95% CI 0.84–1.00), very preterm birth (RR 0.88, 95% CI 0.80–0.96), and very low birth weight (RR 0.89, 95% CI 0.81–0.98) compared with partner sperm. However, donor sperm conceptions exhibited increased risks of preeclampsia (RR 1.35, 95% CI 1.06–1.74) as well as pregnancy-induced hypertension (RR 1.19, 95% CI 1.05–1.36). For other outcomes, including gestational diabetes mellitus, ectopic pregnancy, placental abruption, placenta previa, large and small for gestational age, preterm birth, high and low birth weight, perinatal death, stillbirth, neonatal death, and congenital anomalies, no significant disparities were observed.

**Conclusions:**

Donor sperm offers improved pregnancy outcomes for severe male infertility or paternally inherited genetic disorders but is linked to elevated risks of preeclampsia and pregnancy-induced hypertension. Additional studies are required to explore potential mechanisms and design specific interventions.

## Graphical Abstract

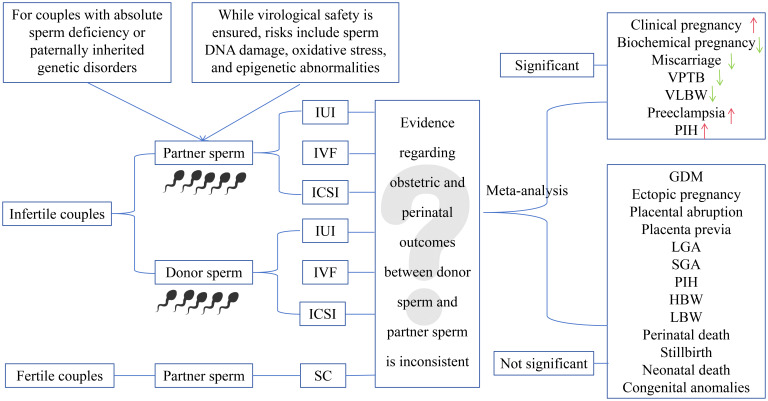

## Introduction

Globally, approximately 15% of couples struggle with infertility, with male factors accounting for 30–40% of these instances (1–3). Assisted reproductive technologies (ART) have revolutionized infertility treatment, and donor sperm has become a crucial approach for couples facing absolute sperm deficiency (e.g., non-obstructive azoospermia) or paternally inherited genetic disorders. Clinically, donor sperm is applied through three approaches: artificial insemination (AID), *in vitro* fertilization (IVF-D), and intracytoplasmic sperm injection (ICSI-D). Among these, ICSI-D is typically reserved for complex cases, such as recurrent IVF failure.

Mandatory six-month cryopreservation of donor sperm effectively eliminates HIV transmission risks but introduces biological challenges. These include ultra-structural sperm damage, mitochondrial dysfunction, and oxidative stress-mediated DNA fragmentation (4–6), which are further exacerbated by laboratory processing techniques such as density gradient centrifugation. These alterations may impair epigenetic reprogramming during fertilization, raising concerns about downstream impacts on blastocyst development and long-term offspring health. These concerns underscore the need for a comprehensive evaluation of donor sperm’s obstetric and perinatal safety profile.

Previous studies comparing donor and partner sperm outcomes remain inconclusive (7–73) due to heterogeneity in study designs, sample sizes, conception methods (e.g., IUI vs. IVF/ICSI), and confounding factors. Although four previous meta-analyses have addressed this topic (74–77), their findings are undermined by several critical limitations (1): reliance on unadjusted estimates (8, 16, 22–26, 28, 29, 31–38, 40–71) (2); the emergence of recent high-quality evidence (7, 12–14, 23, 24, 26–36) (3); the omission of studies that should have been included based on the study period (37–45) (4); incomplete outcome assessment, particularly for clinical pregnancy, biochemical pregnancy, and very high birth weight(VHBW); and (5) language bias due to the exclusion of Chinese studies (29, 31, 33, 35, 36, 40, 41, 43–47).

To address these limitations, we presented the first and largest meta-analysis to date, synthesizing both unadjusted (8, 16, 22–26, 28, 29, 31–38, 40–71) and adjusted data (7–9, 11–14, 17–20, 27, 30, 39, 72, 73) from 64 studies. By systematically evaluating 21 obstetric and perinatal outcomes, we aimed to provide robust evidence to guide clinical decision-making for couples considering the use of donor sperm in ART, while also identifying areas requiring further investigation to optimize maternal and neonatal outcomes.

## Materials and methods

Following the Preferred Reporting Items for Systematic Reviews and Meta-analysis (PRISMA) 2020 guidelines (Supplementary PRISMA checklist), this systematic review and meta-analysis was prospectively registered on PROSPERO (CRD42024568737).

### Literature search

A comprehensive search was conducted across six electronic databases, including PubMed, Embase, Web of Science, Scopus, Wanfang, and China National Knowledge Infrastructure (CNKI), to identify articles published until December 2024. No language or publication date restrictions were applied to minimize selection bias. Detailed search strategies utilized in each database are provided in **Supplementary Data**.

### Outcomes

The outcomes of interest were categorized into three domains: pregnancy outcomes, pregnancy complications, and perinatal outcomes. Pregnancy outcomes included clinical pregnancy (verified by detecting an intrauterine gestational sac), ectopic pregnancy, biochemical pregnancy (defined by serum hCG levels of ≥5 IU/L without ultrasound confirmation), and miscarriage (occurring before 20 weeks of gestation). Pregnancy complications involved pregnancy-induced hypertension (PIH), gestational diabetes mellitus (GDM), preeclampsia (blood pressure ≥140/90 mmHg and proteinuria ≥300 mg/24h), placental abruption, and placenta previa. Perinatal outcomes focused on adverse birth events, including preterm birth (PTB, occurring before 37 weeks’ gestation), very preterm birth (VPTB, occurring before 32 weeks’ gestation), low birth weight (LBW, < 2500 g), high birth weight (HBW, > 4000 g), large for gestational age (LGA, birth weight > 90th percentile), VHBW (> 4500 g), very low birth weight (VLBW, < 1500 g), small for gestational age (SGA, birth weight >10th percentile), stillbirth, neonatal death, perinatal death, and congenital anomalies.

### Inclusion and exclusion criteria

To be deemed eligible for inclusion, studies needed to satisfy the following conditions (1): reporting ≥1 predefined outcome comparing donor and partner sperm, regardless of conception method (2); reporting adjusted/crude relative risks (RR)/odds ratio (OR) and their 95% confidence intervals (CIs) (3); availability of peer-reviewed full texts.

The criteria for exclusion included the following (1): studies that were literature reviews, conference abstracts, case reports (2); studies conducted on non-human subjects (3); studies involving mixed donor gametes (e.g., donor oocytes) (4); duplicate datasets (5); studies lacking sufficient data to calculate effect sizes; and (6) studies comparing different ART methods without stratifying by sperm source.

### Study selection

Retrieved studies were managed using EndNote X8. After removing duplicate records, two authors independently screened each study for eligibility based on title, abstract, or full text. Any disagreements were addressed by either discussion or by seeking input from a third reviewer. All qualifying articles were incorporated into this study without consideration of quality scores, recognizing that even studies with methodological weaknesses may provide valuable evidence (78, 79).

### Data extraction

Two authors independently conducted data extraction from the selected articles, capturing the following information: first author, publication year, study period, study design, country, occurrence of multiple births, model of conception, sample size, controlled confounders, and outcomes. When adjusted estimates were available, they were preferred; otherwise, crude estimates were utilized. Any discrepancies that arose during data extraction were addressed by either discussion or by seeking input from a third reviewer.

### Quality assessment

Two researchers independently evaluated the quality of the selected articles utilizing the Joanna Briggs Institute (JBI) critical appraisal checklist (80). Any disagreements were addressed by either discussion or by seeking input from a third reviewer. For each item, a score was assigned as follows: 0 for “no”, 1 for “unclear”, and 2 for “yes”. Studies were classified into three quality categories: high quality (70% or above), medium quality (between 40% and 70%), and low quality (below 40%) based on summary scores (the total score divided by the maximum achievable score) (81).

### Statistical methods

The formula RR=OR/[(1-P0)+(P0*OR)] was used to convert the OR to the RR, where P0 represents the outcome incidence rate in the control group (82). To transform the 95% CIs, the following formula was applied: SElog(RR)=SElog(OR)×log(RR)/log(OR) (83). The I^2^ statistic was utilized to evaluate the extent of heterogeneity among studies, and a value exceeding 50% denotes notable heterogeneity. To explore possible sources of heterogeneity, subgroup analyses were carried out by stratifying the data based on whether confounding factors were adjusted (yes or no), the model of conception (such as donor sperm IUI vs. partner sperm IUI), and location (Asian vs. non-Asian). To assess heterogeneity between subgroups, univariate meta-regression under a random-effects model was performed using R software (version 4.5.1) to obtain the corresponding *P* values. Sensitivity analyses, including itemized exclusions and effect-size weighting, were utilized to verify the stability of the findings while funnel plots and Egger’s regression test were applied to detect potential publication bias. Owing to clinical and methodological heterogeneity, crude and adjusted RRs were synthesized employing random-effects models. The criteria devised by the Grading of Recommendations, Assessment, Development, and Evaluation (GRADE) Working Group enabled us to assess the overall evidence quality regarding the relationship between various sperm sources and obstetric as well as perinatal outcomes.

## Results

### Characteristics of eligible studies

**Figure 1:**
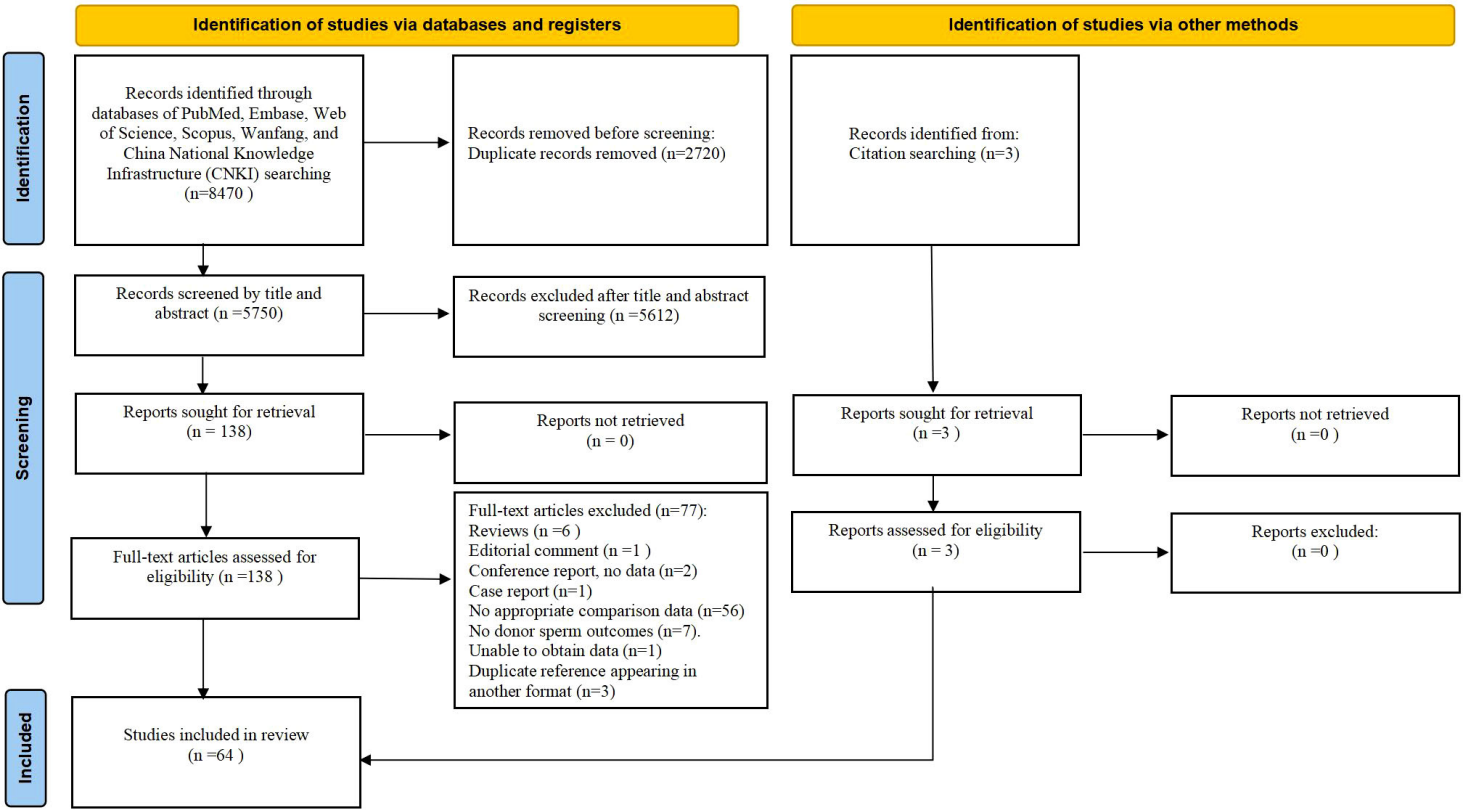
PRISMA flowchart of literature screening.

Initial database searches and reference screening identified 8470 articles. After eliminating duplicates and examining titles/abstracts, 141 articles were chosen for full-text assessment. Of these, 77 articles were excluded according to predefined criteria (**Supplementary Table S1**). The final analysis included 64 studies (**Figure 1**). **Table 1** presents the main characteristics of those eligible articles. Among these, twenty were multicenter (7–11, 13, 17–19, 25, 28, 30, 42, 48, 52, 57, 60, 65, 68, 71), while the remaining were conducted at either single-center (12, 14, 16, 20, 22–24, 26, 27, 29, 31–38, 40, 41, 43–47, 49–51, 53–56, 58, 59, 61, 63, 64, 66, 67, 69, 70) or dual-center settings (62, 72, 73). Adjusted confounders for obstetric/perinatal outcomes were reported in 16 studies (7–9, 11–14, 17–20, 27, 30, 39, 72, 73), whereas others provided unadjusted estimates (8, 16, 22–26, 28, 29, 31–38, 40–71). Quality assessment using the JBI critical appraisal tool revealed 47 studies (73.44%) as medium-quality and 17 studies (26.56%) as high-quality. Most studies had adequate selection of participants and clearly distinguished between donor and partner sperm conception, but control of confounding factors and reporting of follow-up were often insufficient (**Supplementary Table S2**). Evidence certainty, assessed using the GRADE criteria, was rated as moderate for biochemical pregnancy, preeclampsia, PIH, VPTB, VLB, and VHBW. Evidence for miscarriage, placenta previa, PTB, HBW, stillbirth, neonatal death, perinatal death, and congenital anomalies was classified as low certainty, while the evidence certainty for the remaining outcomes was deemed very low (**Supplementary Table S3**).

**Table 1:** Characteristics of eligible studies.

Study (year)	Country	Study period	Study design	Included multiple births	Model of conception (donor vs. partner)	N (donor/partner)	Controlled confounders	Outcomes variable
Adams et al., 2017 (11)	Australia	1986-2002	Multicenter retrospective cohort	Yes	IUI vs. SC	476 births/ 297280 births	Maternal age, parity, ethnicity, socioeconomic quartile, and fetal sex	PTB, VPTB, LBW, VLBW, SGA, LGA
Allen et al., 2022 (13)	UK	1991-2016	Multicenter retrospective cohort	Yes	IVF vs. IVF	9541 births/ 166568 births	Maternal age, history of previous pregnancy, cause of infertility, year of treatment, fresh or frozen	PTB, VPTB, VLBW, LBW, HBW, VHBW, congenital anomaly
Allen et al., 2023 (7)	UK	1991-2016	Multicenter retrospective cohort	Yes	IVF vs. IVF	17634 pregnancies/ 308920 pregnancies	Maternal age, pregnancy history, number of embryos transferred, IVF or ICSI, fresh or frozen cycle, and causes of infertility and year of treatment	BP, miscarriage, EP, stillbirth
Alorf et al., 2024 (27)	Canada	2008-2018	Single-center retrospective cohort	Yes	IUI vs. IUI	Not explained	Patient age at treatment, number of previous pregnancies, daily gonadotrophin dose in the last IVF cycle, number of follicles≥14 mm in the first IUI cycle at the time of trigger, number of failed IVF cycles, use of donor or partner sperm	CP
Azem et al., 1994 (37)	Israel	Before 1993	Single-center retrospective cohort	Yes	IVF vs. IVF	52 cycles/ 259 cycles	None	CP
Bai et al., 2020 (28)	China	2016	Multicenter retrospective cohort	Yes	IUI vs. IUI	34504 cycles/126872 cycles	None	CP, congenital anomaly
Bortoletto et al., 2020 (12)	USA	2008-2018	Single-center retrospective cohort	Yes	IVF vs. IVF	307 cycles/ 3603 cycles	Maternal age, the number of embryos transferred, and the developmental stage of the embryo at the time of transfer	CP, BP, miscarriage, EP, stillbirth
Bu et al. 2016 (20)	China	2009-2015	Single-center retrospective cohort	Yes	IUI vs. IUI	1389 cycles/904 cycles	Tubal factor, type of cycle	EP
Chen et al., 2018 (22)	China	2012-2015	Single-center retrospective cohort	No	IUI vs. IUI	173 pregnancies/ 304 pregnancies	None	Miscarriage, EP, PIH, PTB, LBW, stillbirth, congenital anomaly
Cheng et al., 2018 (29)	China	2015-2017	Single-center retrospective cohort	Yes	ICSI vs. ICSI	38 cycles/392 cycles	None	CP, miscarriage
Davies et al., 2012 (72)	Australia	1986-2002	Two-center retrospective cohort	No	IUI vs. SC	428 births/ 293314 births	Maternal age, parity, fetal sex, year of birth, maternal race or ethnic group, maternal country of birth, maternal conditions in pregnancy, maternal smoking during pregnancy, socioeconomic status, maternal and paternal occupation	Congenital anomaly
Dong et al., 2011 (38)	China	1998-2010	Single-center retrospective cohort	Yes	IUI vs. IUI	1828 cycles/4532 cycles	None	CP
Dunietz et al. 2017 (19)	USA	2000-2010	Multicenter retrospective cohort	No	IVF/ICSI vs. IVF/ICSI	217 births/ 5857 births	Parity, age, race/ethnicity, education level, state of residence and delivery year	PTB
de Mouzon et al., 2007 (48)	France	1996-2003	Multicenter retrospective cohort	No	IVF/ICSI vs. IVF/ICSI	1104 pregnancies/ 32662 pregnancies	None	Miscarriage, EP
Esteves et al., 2014 (49)	Brazil	Before 2014	Single-center retrospective cohort	Yes	IVF/ICSI vs. IVF/ICSI	40 cycles/297 cycles	None	CP, miscarriage, perinatal death, congenital anomaly
Frank et al., 2022 (23)	Canada	2009-2018	Single-center retrospective cohort	Yes	IUI vs. IUI	175 cycles/ 1421 cycles	None	CP
Gao et al., 2022 (14)	China	2015-2019	Single-center retrospective cohort	Yes	IVF/ICSI vs. IVF/ICSI	1559 pregnancies/ 4677 pregnancies	Maternal age, BMI, years of infertility, basic FSH, LH, and E2 levels, partner’s age, infertility type, COH protocol, infertility reason, sperm quality before IVF/ICSI, and transferred embryo quality	CP, BP, PTB, EP, miscarriage, GDM, PE, PP, PA
Gaudoin et al., 2003 (50)	UK	1993-1997	Single-center retrospective cohort	No	IUI vs. IUI/SC	35 pregnancies/ 109408 pregnancies	None	PE, PTB, LBW
Gerkowicz et al., 2018 (18)	UMISCARRIAGE	2010-2014	Multicenter retrospective cohort	No	IVF/ICSI vs. IVF/ICSI	22619 cycles/ 414950 cycles	Maternal age, gravidity, parity, number of prior ART cycles, causes of infertility, stimulation type, hyperstimulation, number of oocytes retrieved, and pre-implantation genetic diagnosis/screening (PGD/PGS)	CP, PTB, LBW
Guo et al., 2017 (46)	China	2012-2016	Single-center retrospective cohort	Yes	ART/ART	270 births/ 234 births	None	Congenital anomaly
Hall et al., 2001 (51)	UK	1991-1998	Single-center retrospective cohort	No	IUI VS IVF	45 pregnancies/ 173 pregnancies	None	PIH, PE
Han et al., 2010 (52)	China	1998-2007	Multicenter retrospective cohort	Yes	IVF/ICSI vs. IVF/ICSI	509 births/ 7998 births	None	Congenital anomaly
Hinduja et al., 2008 (53)	India	2003	Single-center Prospective study	No	ICSI vs. ICSI	10 pregnancies/ 5 pregnancies	None	Miscarriage
Hoy et al., 1999 (73)	Australia	1982-1995	Two-center retrospective cohort	Yes	IUI vs. SC	1552 pregnancies/7717 pregnancies	Maternal age and parity	PE, PTB, LBW, congenital anomaly, perinatal death, stillbirth, neonatal death
Huang et al., 2016 (54)	China	2006-2012	Single-center retrospective cohort	Yes	IUI vs. SC	1406 births/ 1014 births	None	LBW, HBW, congenital anomaly
Kamath et al., 2018 (17)	UK	1991-2011	Multicenter retrospective cohort	No	IVF/ICSI vs. IVF/ICSI	4523 births/ 91264 births	Age of women, duration of treatment, causes of infertility, previous live birth, number of embryos transferred, ICSI, and initial multiple pregnancy that lead to singleton live births	PTB, VPTB, LBW, VLBW, HBW
Kennedy et al., 2019 (30)	Australia	2009-2017	Multicenter retrospective cohort	Yes	IVF vs. IVF	1435 pregnancies/ 13191 pregnancies	Maternal age, BMI. and fertilization via ICSI	PIH/PE
Kyrou et al., 2010 (55)	Belgium	1999-2006	Single-center retrospective cohort	Yes	IUI vs. IUI	438 pregnancies/ 275 pregnancies	None	PE
Laivuori et al., 1998 (56)	Finland	Before 1998	Single-center retrospective cohort	Yes	IUI/IVF vs. IUI/IVF/SC	73 pregnancies/ 50 pregnancies	None	PIH, PE, SGA, stillbirth
Lansac et al., 1997 (91)	France	1987-1994	Multicenter Prospective cohort	Yes	IUI vs. IUI	8943 births/ 13631 births	None	PTB, LBW, stillbirth, congenital anomaly
Liu et al., 2017 (47)	China	2013-2015	Single-center retrospective cohort	Yes	IVF/ICSI vs. IVF/ICSI	127 births/ 119 births	None	HBW, LBW, VLBW, congenital anomaly
Luke et al., 2016 (39)	UMISCARRIAGE	2004-2008	Multicenter retrospective cohort	Yes	IVF/ICSI vs. IVF/ICSI	283 pregnancies/ 7563 pregnancies	Maternal and paternal age, race and ethnicity, and education; diagnoses; maternal preexisting medical conditions, plurality at six weeks	PIH, GDM, PTB, LBW, SGA, LGA, congenital anomaly
Malchau et al., 2014 (9)	Denmark	2007-2012	Multicenter retrospective cohort	No	IUI vs. IUI	1881 births/ 4208 births	Year of birth, parity, maternal age, child gender, BMI, smoking, elective cesarean section, and induction of labor	LBW, PTB, SGA, LGA
Ni et al., 2022 (31)	China	2016-2019	Single-center retrospective cohort	Yes	IVF vs. IVF	129 cycles/ 111 cycles	None	CP, miscarriage, PTB, LBW, HBW
Oehninger 1998 (58)	USA	1994-1997	Single-center retrospective cohort	No	IVF/ICSI vs. IVF/ICSI	105 cycles/ 952 cycles	None	CP, miscarriage
Plasencia et al., 2004 (59)	Spain	1999-2002	Single-center retrospective cohort	No	IUI vs. IUI	67 pregnancies/ 252 pregnancies	None	Miscarriage
Prados et al., 2017 (60)	Spain	2012-2013	Multicenter retrospective cohort	No	IUI vs. IUI	2330 cycles/ 4263 cycles	None	Miscarriage, EP
Robinson et al., 1993 (16)	UK	Before 1992	Single-center retrospective cohort	Yes	IVF vs. IVF	31 pregnancies/ 20 pregnancies	None	Miscarriage
Ruiter-Ligeti et al., 2020 (32)	Canada	2011-2018	Single-center retrospective cohort	Yes	IUI vs. IUI	49 cycles/ 276 cycles	None	CP
Saavedra-Saavedra et al., 2012 (61)	Spain	2000-2009	Single-center retrospective cohort	Yes	IUI vs. IUI	164 pregnancies/ 264 pregnancies	None	PE, PIH
Salha et al., 1999 (62)	UK	1992-1997	Two-center retrospective cohort	Yes	IUI vs. IUI	29 pregnancies/ 27 pregnancies	None	PE, PIH
Scarselli et al., 2018 (63)	Italy	2014-2016	Single-center retrospective cohort	No	ICSI vs. ICSI	26 cycles/40 cycles	None	CP, miscarriage, BP, IP
Smith et al.1997 (64)	Canada	Before 1997	Single-center retrospective cohort	Yes	IUI vs. IUI	37 pregnancies/ 44 pregnancies	None	PE, stillbirth
Su et al., 2014 (40)	China	2012-2013	Single-center retrospective cohort	Yes	IVF vs. IVF	202 cycles/202 cycles	None	CP, miscarriage
Sun et al., 2022 (24)	China	2011-2020	Single-center retrospective cohort	Yes	ICSI vs. ICSI	37 cycles/ 2102 cycles	None	CP, BP, miscarriage
Thapar et al., 2007 (65)	UK	2004-2006	Multicenter retrospective cohort	Yes	IVF/ICSI vs. IVF/ICSI	170 pregnancies/ 378 pregnancies	None	PIH, GDM, LBW
Varma et al., 1987 (66)	UK	1983-1985	Single-center retrospective cohort	Yes	IUI vs. IUI/IVF/SC	72 pregnancies/ 8321 pregnancies	None	Miscarriage, EP, PIH, GDM, PP, PA, PTB, VPTB, LBW, VLBL, SGA, HBW, stillbirth, neonatal death, congenital anomaly
Verp et al., 1983 (67)	USA	1976-1980	Single-center retrospective cohort	Yes	IUI vs. IVF/ICSI	121 births/ 426 births	None	Congenital anomaly
Wang et al., 2019 (33)	China	2009-2017	Single-center retrospective cohort	Yes	IUI/IVF/ICSI vs. IUI/IVF/ICSI	2177 births/ 15166 births	None	Congenital anomaly
Warnes et al., 1998 (68)	Australia and New Zealand	1979-1993	Multicenter retrospective cohort	No	IVF/ICSI vs. IVF/ICSI	980 pregnancies/ 10340 pregnancies	None	EP, miscarriage
Xu et al., 2014 (41)	China	2004-2013	Single-center retrospective cohort	Yes	IVF/ICSI vs. IVF/ICSI	63 cycles/ 87 cycles	None	CP, miscarriage, congenital anomaly
Yan et al., 2011 (42)	China	2004-2008	Multicenter retrospective cohort	Yes	IUI vs. IUI	1572 births/ 873 births	None	Congenital anomaly
Yan et al., 2015 (43)	China	2012-2013	Single-center retrospective cohort	Yes	IUI vs. IUI	162 pregnancies/ 146 pregnancies	None	PTB, miscarriage, PIH, GDM, LBW, HBW, congenital anomaly
Yang et al., 2021 (34)	China	2015-2017	Single-center retrospective cohort	No	IUI vs. IUI	658 pregnancies/ 413 pregnancies	None	Miscarriage, PTB, LBW, HBW, congenital anomaly
Yovich et al., 1988 (69)	Australia	1980-1985	Single-center retrospective cohort	No	IUI vs. IUI	116 pregnancies/ 76 pregnancies	None	Miscarriage, EP
Yu et al., 2018 (8)	USA	2012-2013	Multicenter retrospective cohort	No	ART vs. ART	2123 pregnancies/ 42799 pregnancies	Maternal age, race, BMI, smoking status, gravidity, history of preterm birth, maximum FSH, blastocyst transfer, total embryo transferred, and etiology of infertility	Miscarriage, PTB, VPTB, LBW, VLBW
Yu et al., 2018b (70)	China	2012-2017	Single-center retrospective cohort	Yes	ICSI vs. ICSI	46 births/ 122 births	None	PTB, LBW, stillbirth
Zamora et al., 2014 (71)	Spain	2010-2011	Multicenter retrospective cohort	No	IUI vs. IUI	1959 pregnancies/ 4477 pregnancies	None	Miscarriage, EP
Zhang et al., 2014 (44)	China	2011-2012	Single-center retrospective cohort	Yes	ICSI vs. ICSI	25 cycles/349 cycles	None	CP
Zheng et al., 2016 (45)	China	2014-2016	Single-center retrospective cohort	Yes	IVF vs. IVF	148 cycles/47 cycles	None	CP, miscarriage
Zhou et al., 2018 (25)	China	2013-2015	Multicenter retrospective cohort	Yes	IUI vs. IUI	4405 cycles/ 21116 cycles	None	CP, miscarriage, PTB, congenital anomaly
Zhu et al., 2021 (35)	China	2016-2020	Single-center retrospective cohort	Yes	ICSI vs. ICSI	32 cycles/81 cycles	None	CP
Zhu et al., 2022 (26)	China	2013-2019	Single-center retrospective cohort	Yes	IVF vs. IVF	287 cycles/ 573 cycles	None	CP, BP, miscarriage, congenital anomaly
Zhu et al., 2024 (36)	China	2011-2021	Single-center retrospective cohort	Yes	IVF/ICSI vs. IVF/ICSI	773 births/ 6153 births	None	PTB, LBW, HBW, congenital anomaly

CP, clinical pregnancy; EP, ectopic pregnancy; BP, biochemical pregnancy; PTB, preterm birth; VPTB, very preterm birth; LBW, low birth weight; VLBW, very low birth weight; HBW, high birth weight; VHBW, very high birth weight; SGA, small for gestational age; LGA, large for gestational age; PIH, pregnancy-induced hypertension; GDM, gestational diabetes mellitus; PE, preeclampsia; PP, placenta praevia; PA, Placental abruption.

### Pregnancy outcomes: donor vs. partner sperm

**Figure 2:**
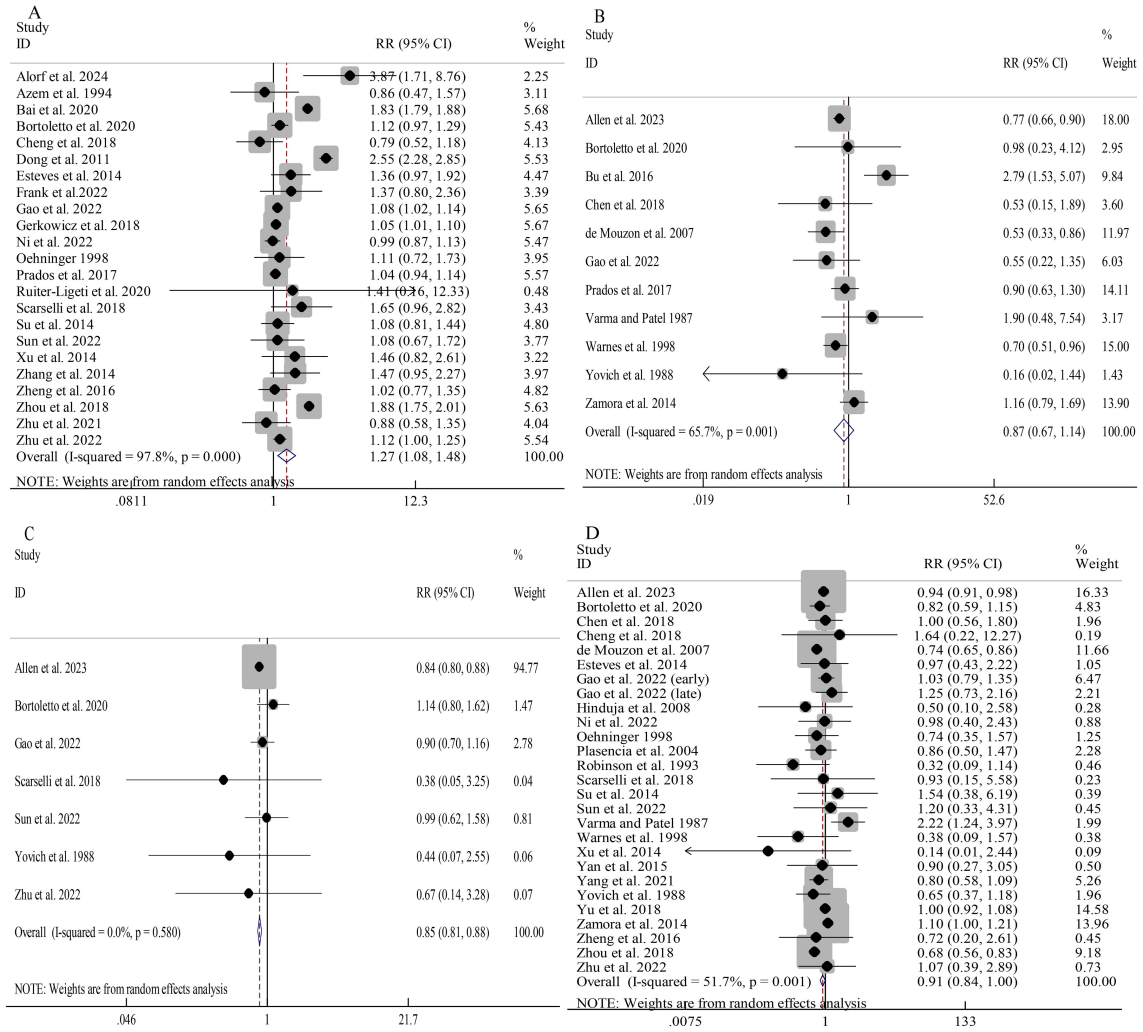
Association between different sperm sources and pregnancy outcomes: clinical pregnancy **(A)**; ectopic pregnancy **(B)**; biochemical pregnancy **(C)**; miscarriage **(D)**.

Donor sperm was linked to a significantly elevated overall clinical pregnancy rate compared to partner sperm (RR 1.27, 95%CI 1.08-1.48; **Figure 2A**). Consistent findings were observed across all subgroups despite notable heterogeneity (I^2^ = 97.8%; **Supplementary Figure S1**). Univariable meta-regression showed that only the model of conception significantly contributed to heterogeneity (QM=16.68, *P*<0.001, R^2^ = 51.24%), while location and adjustment for confounding factors had no significant effects (all *P*>0.05) (**Supplementary Table S4**).

Donor sperm did not significantly differ from partner sperm in overall risk of ectopic pregnancy (RR 0.87, 95%CI 0.67-1.14; **Figure 2B**). Subgroup analyses indicated a reduced ectopic pregnancy risk in donor sperm cycles involving IVF/ICSI (RR 0.74, 95%CI 0.65-0.84) as well as non-Asian cohorts (RR 0.80, 95%CI 0.66-0.98) (**Supplementary Figure S2**). Univariable meta-regression indicated that none of the examined moderators significantly contributed to heterogeneity (all *P*>0.05), although the model of conception showed a trend toward significance (QM=5.45, P=0.065, R^2^ = 58.56%) (**Supplementary Table S4**).

Donor sperm conception was associated with a significantly lower overall risk of biochemical pregnancy than partner sperm conception (RR 0.85, 95%CI 0.81-0.88; I^2^ = 0.0%; **Figure 2C**). While the overall miscarriage risk showed a trend toward reduction with donor sperm (RR 0.91, 95%CI 0.84-1.00; **Figure 2D**), substantial heterogeneity (I^2^ = 51.7%) prompted prespecified subgroup analyses. No significant associations were observed in unadjusted analyses, IUI vs IUI, and Aisan cohorts, while findings from other subgroups were consistent with the overall results (**Supplementary Figure S3**). Univariable meta-regression indicated that only the model of conception significantly contributed to heterogeneity (QM=7.80, *P*=0.020, R^2^ = 11.31%), whereas location and adjusted confounding factors showed no significant effects (all *P*>0.05) (**Supplementary Table S4**).

### Pregnancy complications: donor vs. partner sperm

**Figure 3:**
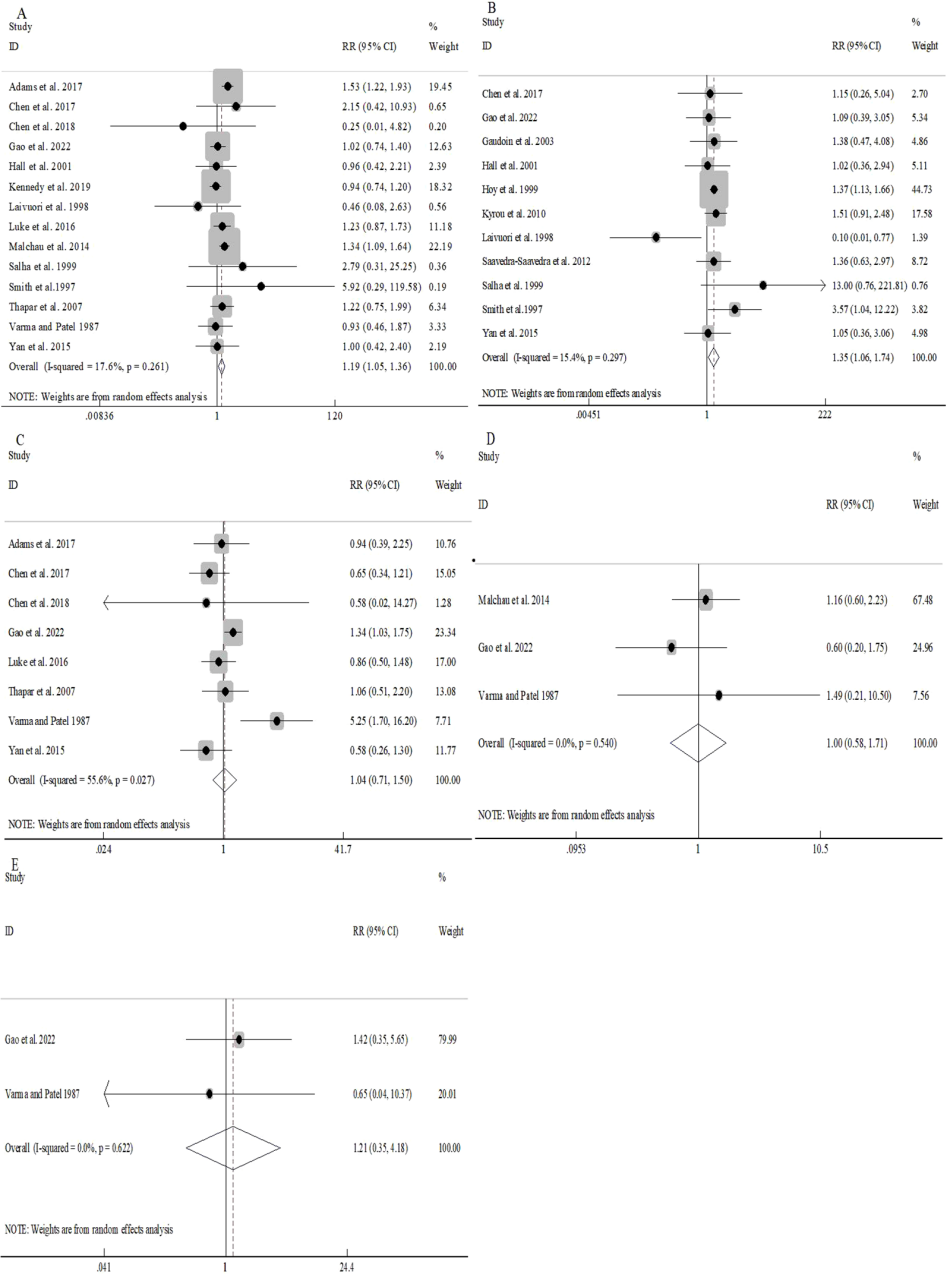
Association between different sperm sources and pregnancy complications: PIH **(A)**; pre-eclampsia **(B)**; GDM **(C)**; placenta praevia **(D)**.

Pooled analyses demonstrated significantly increased risks of preeclampsia (RR 1.35, 95%CI 1.06-1.74; **Figure 3B**) as well as PIH (RR 1.19, 95%CI 1.05-1.36; **Figure 3A**) in donor sperm conceptions, with minimal between-study heterogeneity (I^2^ = 17.6% and I^2^ = 15.4%, respectively).

No notable discrepancies were detected in the overall risk of GDM between donor and partner sperm conception (RR 1.04, 95%CI 0.71-1.50; **Figure 3C**). However, moderate heterogeneity (I^2^ = 55.6%) prompted stratified analyses, which revealed consistent patterns (**Supplementary Figure S4**). Univariable meta-regression indicated that location explained a substantial proportion of heterogeneity (R^2^ = 60.81%, *P*=0.064), but was not statistically significant. Other moderators accounted for minimal heterogeneity (all R^2^≈0, *P*>0.05) (**Supplementary Table S4**).

Similarly, the overall risks of placenta previa (RR 1.00, 95%CI 0.58-1.71; **Figure 3D**) as well as abruption (RR 1.21, 95%CI 0.35-4.18; **Figure 3E**) were comparable between groups with negligible heterogeneity (I^2^ = 0.0% for both).

### Perinatal outcomes: donor vs. partner sperm

**Figure 4:**
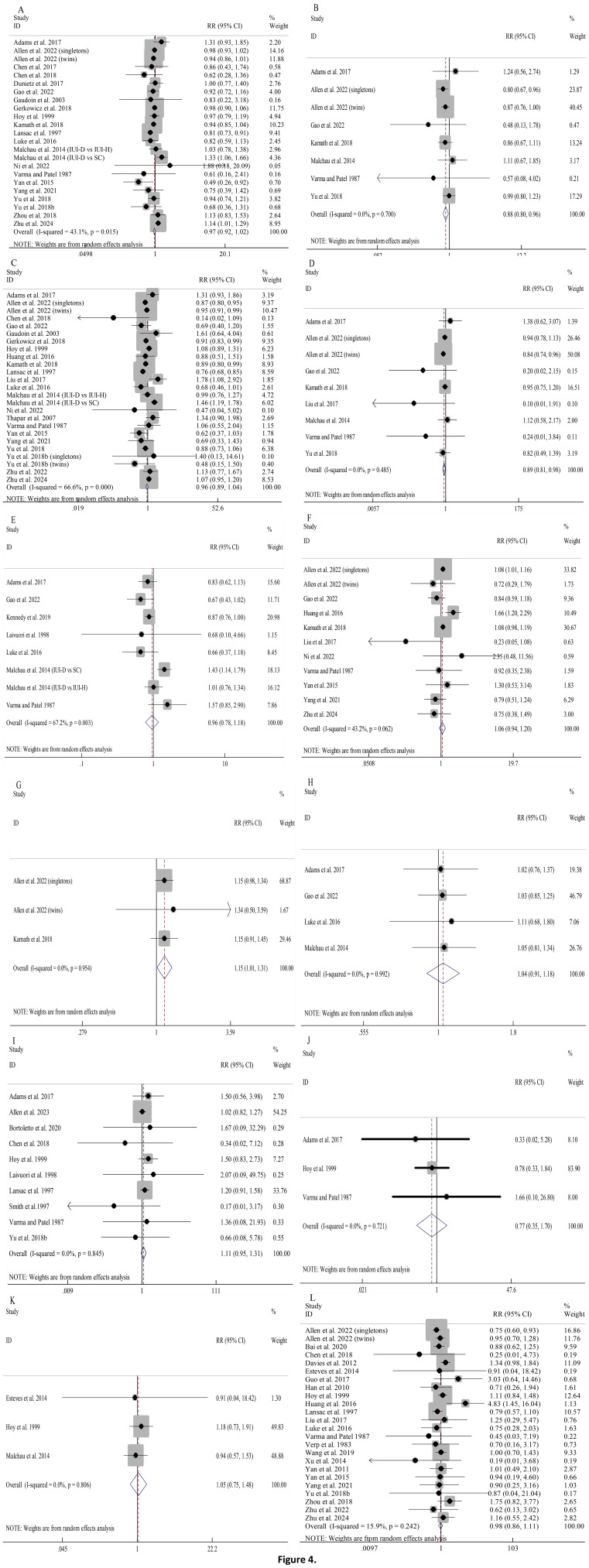
Association between different sperm sources and perinatal outcomes: PTB **(A)**; VPTB **(B)**; LBW **(C)**; VLBW **(D)**; SGA **(E)**; HBW **(F)**; VHBW **(G)**; LGA **(H)**; stillbirth **(I)**; neonatal death **(J)**; perinatal death **(K)**; congenital anomalies **(L)**.

Pooled analyses indicated that using donor sperm had comparable risks of PTB, HBW, LGA, neonatal death, stillbirth, congenital anomalies, and perinatal death when compared to partner sperm, with very low statistical heterogeneity across these outcomes (**Figures 4A, F, H–L**).

Notably, compared to partner sperm, pregnancies resulting from donor sperm exhibited significantly lower risks of VPTB (RR 0.88, 95%CI 0.80-0.96; **Figure 4B**) as well as VLBW (RR 0.89, 95%CI 0.81-0.98; **Figure 4D**). Furthermore, between-study heterogeneity was minimal (I^2^ = 0.0% for both).

The overall risks of LBW and SGA were comparable between donor and partner sperm conceptions (**Figures 4C, E**). However, substantial heterogeneity (I^2^ = 66.6% and I^2^ = 67.2%, respectively) prompted prespecified subgroup analyses, which were largely consistent with the overall results (**Supplementary Figures S5**, **S6**). Univariable meta-regression indicated that, for LBW, only model of conception was a significant moderator (QM=17.623, *P*=0.002, R^2^ = 76.56%), while for SGA, none of the examined moderators significantly explained the heterogeneity among studies (**Supplementary Table S4**).

### Sensitivity analysis

Sensitivity analyses using itemized exclusion demonstrated the robustness of pooled estimates for most outcomes. However, exclusion of the study by Allen et al. (7) eliminated the observed association between sperm sources and biochemical pregnancy risk (*P*=0.658, **Supplementary Figures S7**-**S9**). To further assess the robustness of our results, we performed additional sensitivity analyses by increasing the weight of each study in turn. The changes in pooled effect size and CIs were minimal (maximum difference: 0.11), and the overall conclusions remained unchanged, indicating robust findings without dominance by any single study (**Supplementary Table S5**).

### Publication bias

No significant publication bias was observed through funnel plots and Egger’s tests (*P*>0.05 for all; **Supplementary Figures S10**-**S12**).

## Discussion

### Main findings

This meta-analysis demonstrated that pregnancies achieved using donor sperm had improved clinical pregnancy rates and reduced risks of biochemical pregnancy, miscarriage, VPTB, and VLBW in comparison to those achieved using partner sperm. However, donor sperm conceptions were associated with elevated risks of PIH and preeclampsia. No notable differences were found between pregnancies achieved with donor sperm and those with partner sperm in other obstetric or perinatal outcomes, including GDM, placenta previa, placental abruption, PTB, or congenital anomalies, etc.

### Comparison with literature

Four previous meta-analyses had investigated obstetric and perinatal outcomes in donor versus partner sperm conceptions (74–77). A meta-analysis conducted in 2017, which included three papers published before 2012, reported no increased risks of LBW, PTB, or congenital anomalies in donor sperm conceptions compared with natural conceptions (74). In 2018, the same research team updated their analysis with three additional publications, identifying elevated risks of LBW and congenital anomalies in donor conceptions but no notable differences in PTB, SGA, LGA, VPTB, and VLBW risks (75). A meta-analysis conducted in 2021, which included 36 publications up to 2019, found that donor sperm conceptions had elevated risks of preeclampsia and SGA, but a decreased risk of ectopic pregnancy, compared to partner sperm conceptions. There were no notable differences in the risks of miscarriage, GDM, PIH, placental abruption, placenta previa, PTB, VPTB, LBW, VLBW, HBW, LGA, perinatal death, neonatal death, stillbirth, or congenital anomalies between donor sperm and partner sperm conceptions (77). In 2022, another meta-analysis, which included 17 studies published up to 2020, concluded that donor sperm conceptions were associated with increased risks of preeclampsia and PIH but found no increased risks of LBW and PTB (76).

In contrast to these earlier studies, our meta-analysis synthesized data from 64 eligible studies, including those published as recently as 2024, to provide a more updated and comprehensive assessment. Unlike previous meta-analyses that only focused on univariate analyses, our study is the first to incorporate both univariate and multivariate analyses, enabling a more robust and accurate evaluation of the relationship between sperm sources and obstetric as well as perinatal outcomes. Furthermore, this study is the first to evaluate and compare the outcomes of clinical pregnancy, biochemical pregnancy, and VHBW between donor sperm and partner sperm conceptions. By incorporating a broader range of studies and outcomes, our analysis offers a more comprehensive and detailed insight into the possible risks and benefits associated with using donor sperm.

### Interpretation of findings

Our meta-analysis reveals both clinically significant benefits and potential risks associated with donor sperm conception. On the one hand, donor sperm use is associated with higher clinical pregnancy rates and reduced risks of biochemical pregnancy, miscarriage, VPTB, and VLBW when compared to partner sperm. These benefits are likely attributable, at least in part, to the rigorous screening procedures implemented for sperm donors. Such screening not only ensures that only healthy individuals with optimal semen parameters can become donors, but also effectively excludes issues such as sexually transmitted infections, genetic diseases, and chromosomal abnormalities through physical examinations and hematological tests, thereby helping to improve early pregnancy outcomes and neonatal health.

On the other hand, an important finding of our study is the significantly elevated risk of PIH and preeclampsia in pregnancies conceived with donor sperm. These hypertensive disorders are associated with significant maternal and neonatal morbidity, underscoring the importance of enhanced surveillance, close monitoring, and early intervention, such as regular blood pressure monitoring and prompt management when necessary. Although the underlying mechanisms are not yet fully understood, potential factors may include immunological incompatibility between mother and fetus (84), as well as possible deleterious effects of sperm cryopreservation on DNA integrity (4, 85, 86) and epigenetic modifications (87–90).

Importantly, our findings did not show an increased risk of several other maternal and perinatal complications, including GDM, placenta previa, placental abruption, LBW, SGA, PTB, HBW, VHBW, neonatal death, stillbirth, congenital anomalies, and perinatal mortality, suggesting that donor sperm conception does not broadly elevate perinatal risk beyond hypertensive disorders. However, sample sizes for rarer outcomes were limited.

### Strengths and weaknesses

This meta-analysis has six strengths. First, our meta-analysis included a large sample size, incorporating 17 recently published studies (7, 12–14, 23, 24, 26–36), as well as studies omitted from previous meta-analyses (37–45). This extensive inclusion enhances the generalizability of the study results and improves statistical power. Second, it is the first meta-analysis to integrate both univariate (n=38 studies) and multivariate (n=26 studies) estimates, allowing for a more comprehensive comparison of obstetric and perinatal outcomes between donor and partner sperm. Incorporating multivariate estimates helps to address potential confounding variables, thereby improving the robustness of the findings. Third, unlike earlier meta-analyses, this study expanded the scope of analysis by adding clinical pregnancy, biochemical pregnancy, and VHBW, offering a broader perspective on ART outcomes. Fourth, our meta-analysis minimized selection bias by including eligible studies published in multiple languages, including Chinese (29, 31, 33, 35, 36, 40, 41, 43–47), English, and other languages. Fifth, the statistical heterogeneity for most outcomes was low, indicating consistency across studies and strengthening the reliability of the pooled estimates. Sixth, sensitivity analyses showed no significant change in the pooled estimates after excluding any individual study, indicating that the findings were stable and not overly influenced by individual studies.

Nevertheless, it is important to acknowledge the presence of five limitations in this meta-analysis. First, as the studies included were observational, the findings are inevitably subject to residual or uncontrolled confounding factors, which limits the ability to draw definitive causal inferences. While multivariate analyses were included to address some confounding, the inherent design limitations of observational studies remain. Second, the loss of follow-up may affect the accuracy of the results. Couples receiving ART often experience psychological pressure, influenced by traditional beliefs and societal pressures, which may lead to interrupted contact with medical institutions and loss of follow-up. Third, there was inconsistency in the definitions of key confounders and outcome variables among the included studies. Since most of the original studies only reported aggregated data and some did not provide detailed definitions of these variables, it was not possible to fully harmonize variable definitions in our analysis. This inconsistency may have introduced additional heterogeneity. Such heterogeneity is inevitable in most meta-analyses synthesizing observational studies. Fourth, due to the limited baseline information in the included studies, some key variables (such as maternal age, duration of sperm cryopreservation, and the proportion of multiple pregnancies) were either insufficiently reported or not accompanied by stratified outcome data. This limited our ability to perform subgroup analyses or meta-regression based on these variables, and thus hindered a more comprehensive exploration of potential sources of heterogeneity. Fifth, according to the GRADE assessment, only 6 of the 20 outcomes were rated as moderate quality, while the remaining 14 were of low or very low quality. This indicates that the overall reliability of the study’s conclusions is limited, and the related results should be interpreted with caution.

### Implications for clinical practice

For couples facing absolute sperm deficiency or paternally inherited genetic disorders, donor sperm is a viable and effective solution, as it can significantly improve pregnancy success rates and reduce the occurrence of various adverse outcomes. However, clinicians must clearly inform patients of the increased risk of PIH and preeclampsia, strengthen prenatal monitoring, and implement necessary preventive measures. For couples without strict indications for donor sperm use, considering the increased risk of hypertensive disorders, conception with partner sperm remains preferable when feasible. Therefore, for these couples, the potential risks and benefits should be carefully weighed. In addition, this meta-analysis indicates the need for further high-quality studies to clarify the mechanisms underlying the increased risk of PIH and preeclampsia in donor sperm conceptions, and to develop targeted interventions for optimizing ART outcomes.

## Conclusions

Donor sperm is a viable and effective solution for male infertility or paternally inherited disorders and should be prioritized for patients with strict medical indications. Due to increased risks of PIH and preeclampsia, careful risk-benefit assessment is necessary for other patients. Enhanced surveillance and counseling can aid in the early detection and management of complications, thereby improving maternal and fetal outcomes. Further research is needed to elucidate the underlying mechanisms and develop targeted interventions.
