# Supplementary material for: Obstetric and perinatal outcomes in pregnancies conceived with donor versus partner sperm: a systematic review and meta-analysis
Source: Front Endocrinol (Lausanne). 2025 Jul 23;16:1590261. doi: 10.3389/fendo.2025.1590261 (PMC12325047; doi:10.3389/fendo.2025.1590261)
Supplement: Supplementary file 2 [file DataSheet1.doc]

**Supplementary data for**

**Obstetric and perinatal outcomes in pregnancies conceived with donor versus partner sperm: a systematic review and meta-analysis**

Junjie Liu^a†*^, Yanpeng Dai^b†^, Zuozhe Song^a^, Xintao Sun^a^, Dongdong Lv^a^, Dehua Zhao^a*^

*^a^Henan Human Sperm Bank,* *the Third Affiliated Hospital of Zhengzhou University, Zhengzhou, China*

*^b^Department of Clinical Laboratory, the Third Affiliated Hospital of Zhengzhou University, Zhengzhou, China*

*Corresponding author: [zdsfyljj@126.com](mailto:zdsfyljj@126.com) (J. Liu) and ([zhaodehua369@163.com](mailto:zhaodehua369@163.com)). (D. Zhao).

^†^These authors have made equal contributions to this article.

The detailed search strategy.

**Table S1.** Excluded studies and corresponding reasons for exclusion.

**Table S2**. The results of quality assessment using the Joanna Briggs Institute (JBI) critical appraisal tools for cohort studies.

**Table S3.** GRADE assessment of the cumulative evidence for the association between different sperm sources and obstetric and perinatal outcomes.

**Table S4.** Results of univariable regression analysis.

**Table S5.** Results of effect weighting analysis.

**Figure S1.** Subgroup analyses for clinical pregnancy based on adjusted confounding factors (A), model of conception (B), location (C).

**Figure S2.** Subgroup analyses for ectopic pregnancy based on adjusted confounding factors (A), model of conception (B), location (C).

**Figure S3.** Subgroup analyses for miscarriage based on adjusted confounding factors (A), model of conception (B), location (C).

**Figure S4.** Subgroup analyses for gestational diabetes mellitus based on adjusted confounding factors (A), model of conception (B), location (C).

**Figure S5****.** Subgroup analyses for low birthweight based on adjusted confounding factors (A), model of conception (B), location (C).

**Figure S6.** Subgroup analyses for small for gestational age based on adjusted confounding factors (A), model of conception (B), location (C).

**Figure S7.** Sensitivity analysis for the association between different sperm sources and pregnancy outcomes: clinical pregnancy (A); ectopic pregnancy (B); biochemical pregnancy (C); miscarriage (D).

**Figure S8.** Sensitivity analysis for the association between different sperm sources and pregnancy complications: pregnancy-induced hypertension (A); pre-eclampsia (B); gestational diabetes (C); placenta praevia (D).

**Figure S9a.** Sensitivity analysis for the association between different sperm sources and perinatal outcomes: preterm birth (A); very preterm birth (B); low birthweight (C); very low birthweight (D); small for gestational age (E); high birth weight (F).

**Figure S9b.** Sensitivity analysis for the association between different sperm sources and perinatal outcomes: very high birth weight (G); large for gestational age (H); stillbirth (I); neonatal death (J); perinatal death (K); congenital anomaly (L).

**Figure S10.** Funnel plots of the association between different sperm sources and pregnancy outcomes: clinical pregnancy (A); ectopic pregnancy (B); biochemical pregnancy (C); miscarriage (D).

**Figure S11.** Funnel plots of the association between different sperm sources and pregnancy complications: pregnancy-induced hypertension (A); pre-eclampsia (B); gestational diabetes (C); placenta praevia (D).

**Figure S12a.** Funnel plots of the association between different sperm sources and perinatal outcomes: preterm birth (A); very preterm birth (B); low birthweight (C); very low birthweight (D); small for gestational age (E); high birth weight (F).

**Figure S12b.** Funnel plots of the association between different sperm sources and perinatal outcomes: very high birth weight (G); large for gestational age (H); stillbirth (I); neonatal death (J); perinatal death (K); congenital anomaly (L).

The detailed search strategy for database 1 (PubMed).


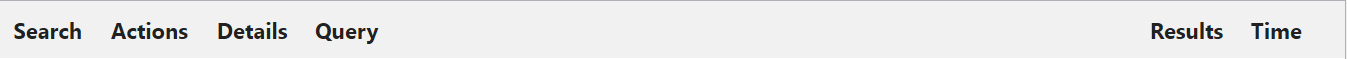

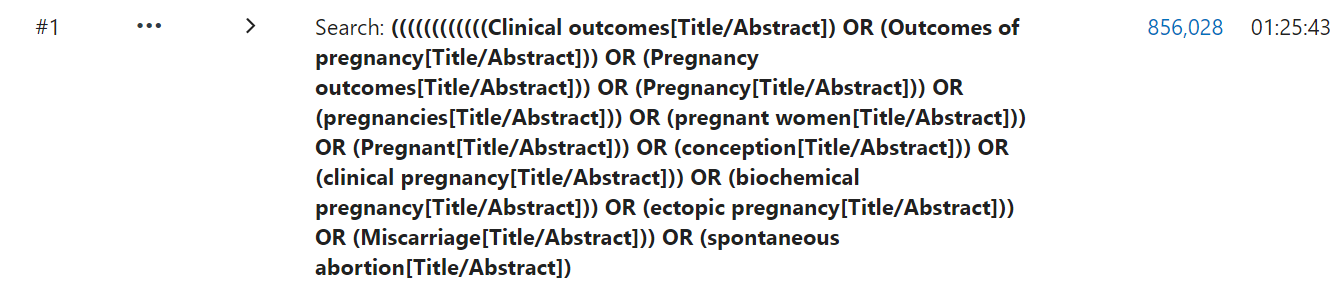

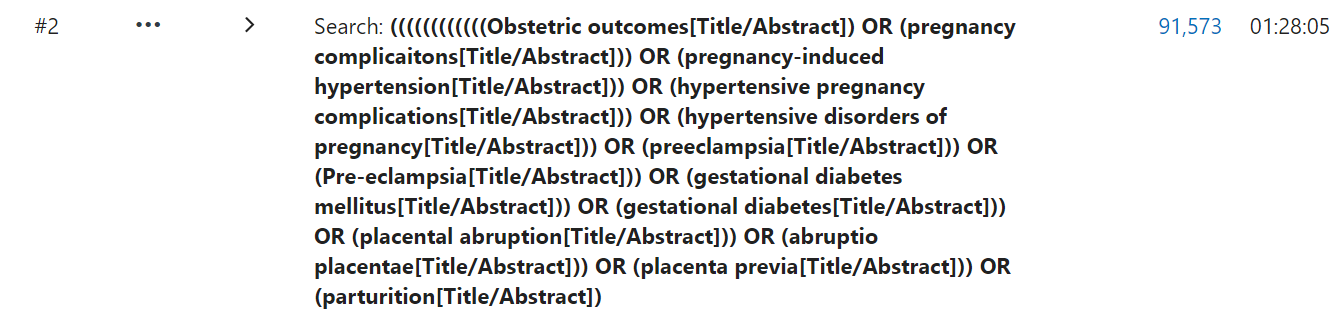

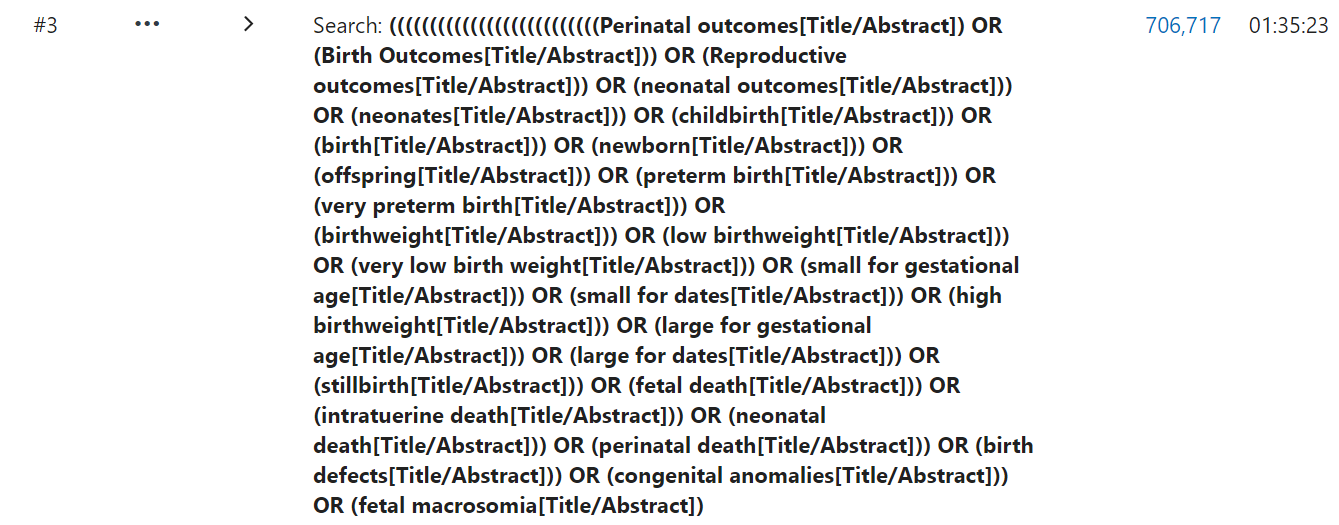

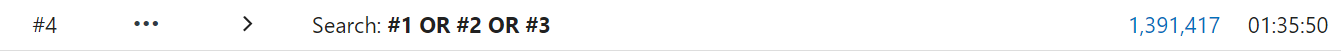

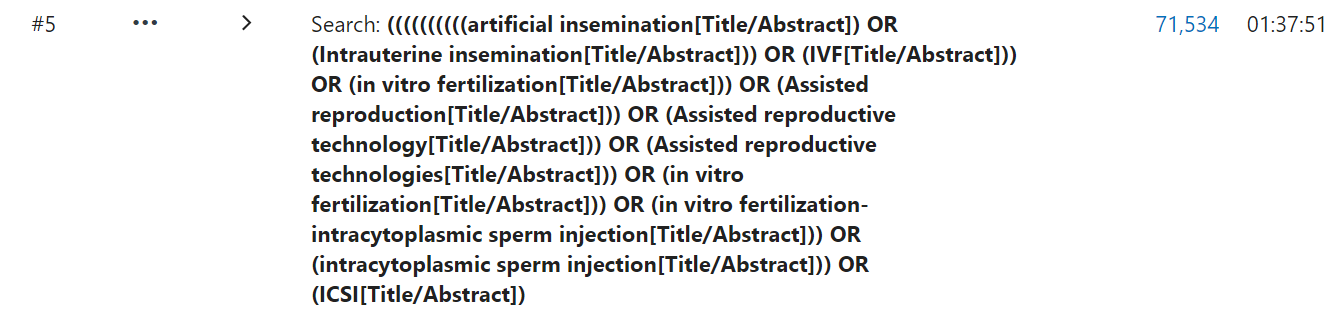

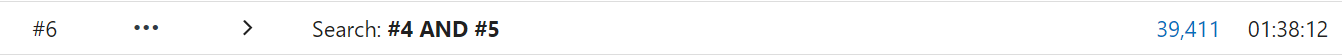

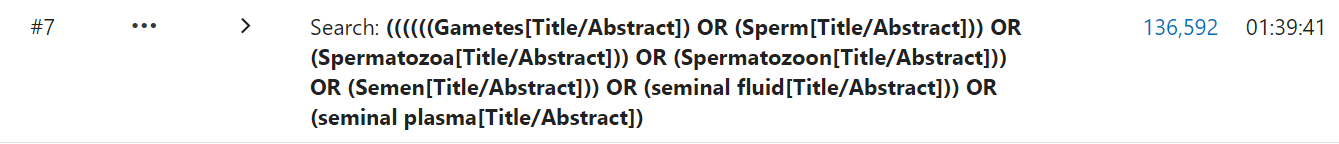

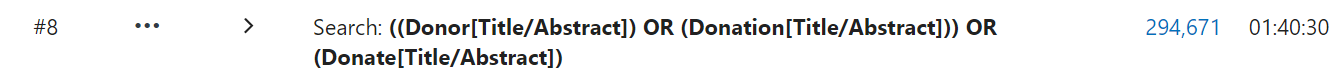

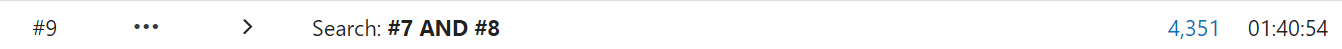

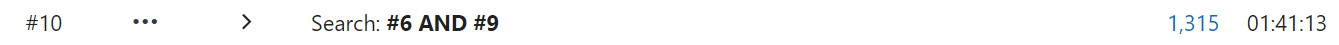


The detailed search strategy for database 2 **(**Web of Science).

、


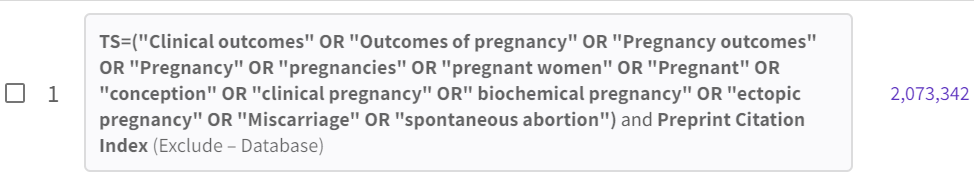

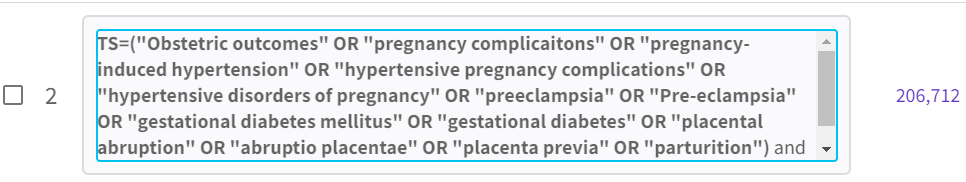

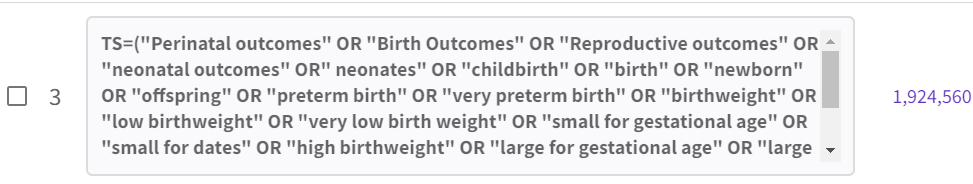

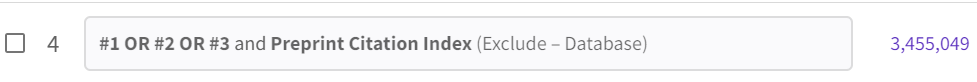

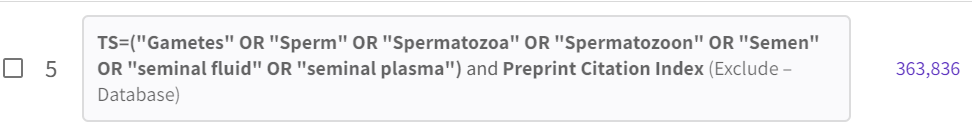

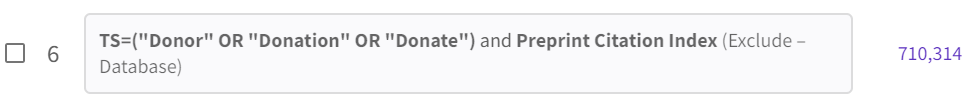

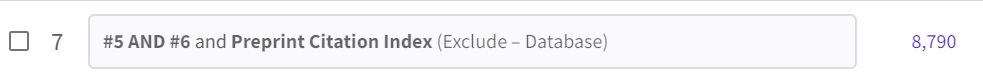

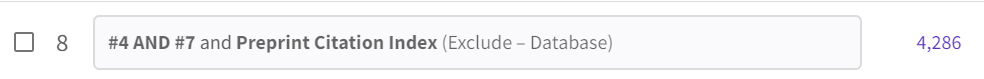

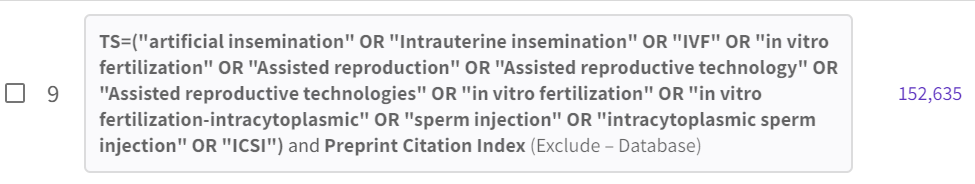

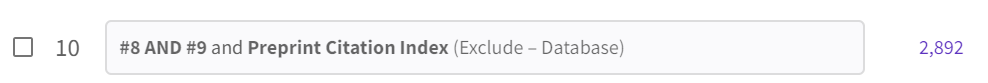


The detailed search strategy for database 3 **(**EMBASE).


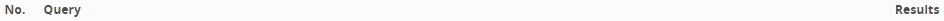

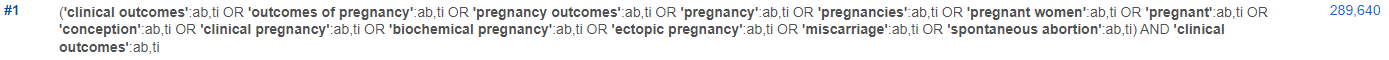

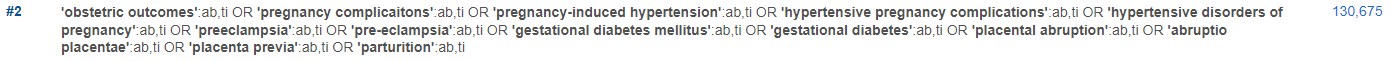

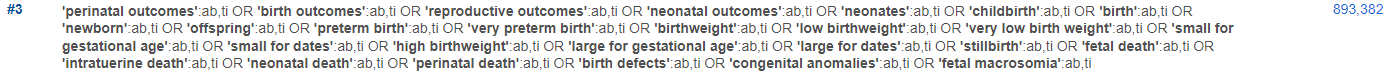

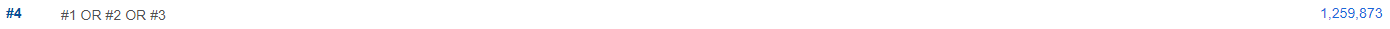

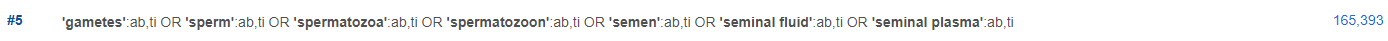

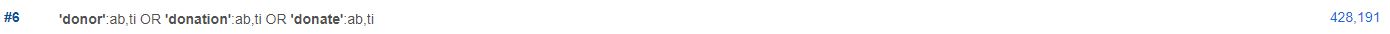

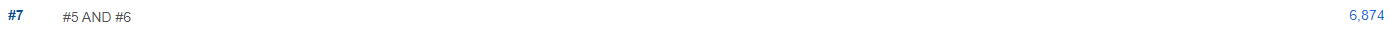

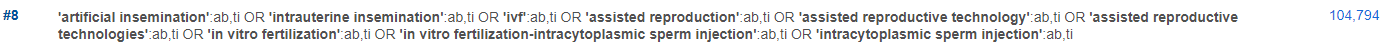

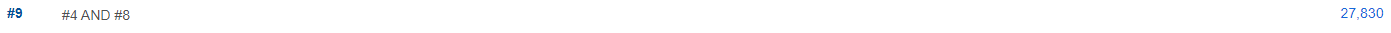

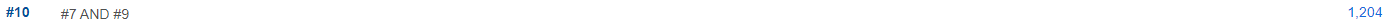

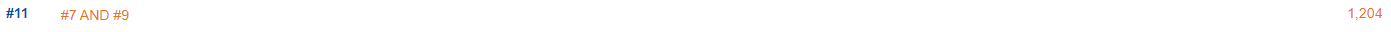


The detailed search strategy for database 4 **(**Scopus).

#1

#2

#3

#1 OR #2 OR #3

#4

#5

#6

#4 AND #5

#7


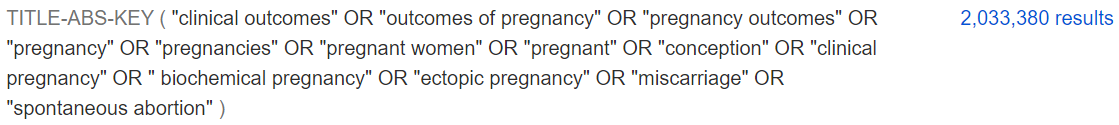

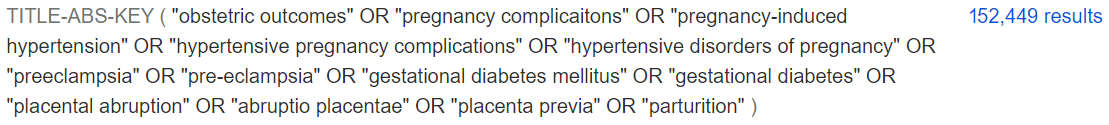

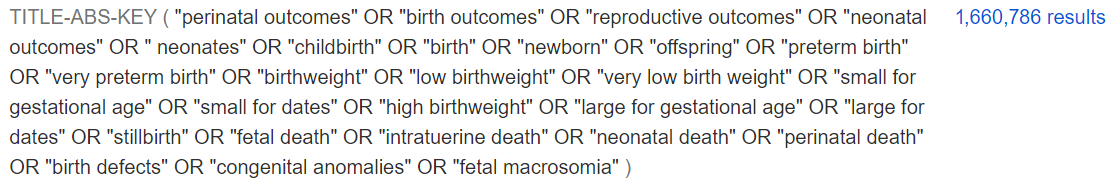

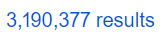

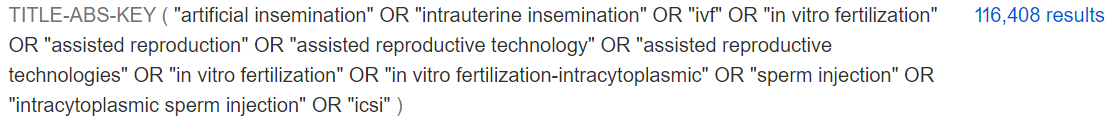

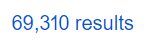

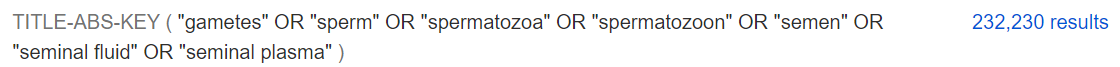


#8


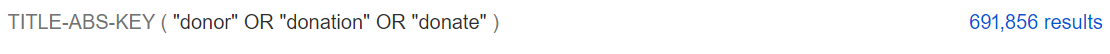


#7 AND #8


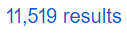


#9

#6 AND #9

#10


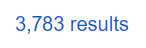


The detailed search strategy for database 5 **(**China National Knowledge Infrastructure databases).
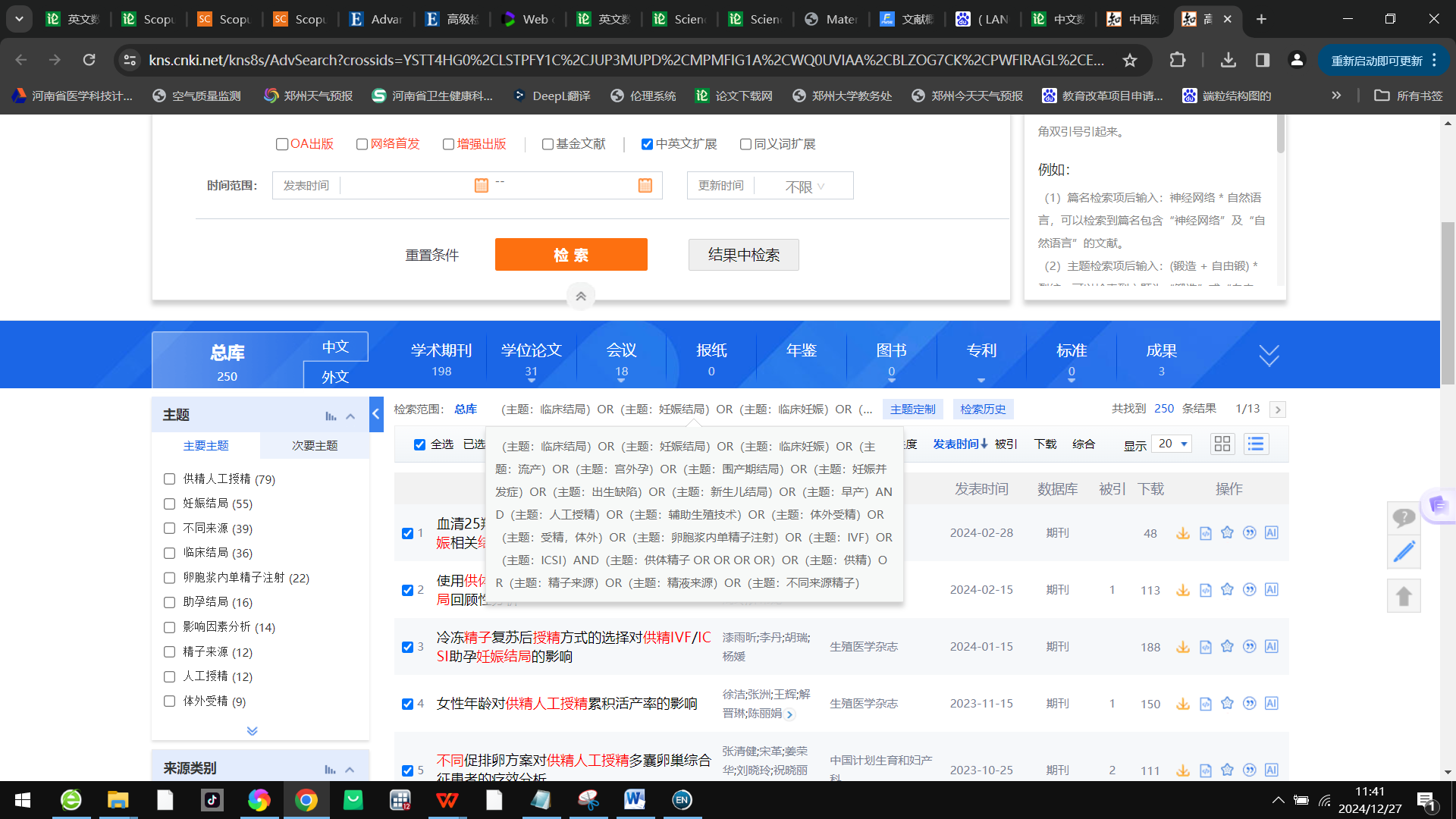


The detailed search strategy for database 6 (Wanfang).

主题：（"临床结局" OR "妊娠结局" OR "临床妊娠" OR "流产" OR "宫外孕" OR "围产期结局" OR "妊娠并发症" OR "出生缺陷" OR "新生儿结局" OR "早产") AND 主题：（"人工授精" OR "辅助生殖技术" OR "体外受精" OR "受精，体外" OR "卵胞浆内单精子注射" OR "IVF" OR "ICSI"）AND 主题：（ "供体精子" OR "供精" OR "精子来源" OR "精液来源" OR "不同来源精子"）

**Table S1.** Excluded studies and corresponding reasons for exclusion.

| **Study** | **Reason for exclusion** |
| --- | --- |
| Adesiyun (2011)^[1]^ | No appropriate comparison data. |
| Alfredsson (1988)^[2]^ | No appropriate comparison data. |
| Allen et al. (2006)^[3]^ | Review. |
| American Fertility Society and SART (1994)^[4]^ | No donor sperm outcomes. |
| Amuzu *et al.* (1990)^[5]^ | No appropriate comparison data. |
| Amuzu and Shapiro (1993)^[6]^ | No appropriate comparison data. |
| Andersen et al. (2008)^[7]^ | No appropriate comparison data. |
| Arcos-Machancoses *et al.* (2015)^[8]^ | Case report. |
| Botchan et al. (2001)^[9]^ | No appropriate comparison data. |
| Bromwich et al. (1978)^[10]^ | No appropriate comparison data. |
| Bustillo and Zarutskie (1996)^[11]^ | Duplicate reference appearing in another format. |
| Bustillo *et al.* (1999)^[12]^ | Duplicate reference appearing in another format. |
| Castillo *et al.* (2019)^[13]^ | No appropriate comparison data. |
| Clarke *et al.* (1997)^[14]^ | No appropriate comparison data. |
| Cottinet et al. (1974)^[15]^ | No appropriate comparison data. |
| de Mouzon et al. (2010)^[16]^ | No appropriate comparison data. |
| Dixon and Buttram (1976)^[17]^ | No appropriate comparison data. |
| Dyer and Kruger (2012)^[18]^ | No appropriate comparison data. |
| Esteves (2002)^[19]^ | Editorial comment. |
| Fang *et al.* (2018)^[20]^ | Unable to obtain data. |
| Ferrara *et al.* (2000)^[21]^ | No appropriate comparison data. |
| Formigli et al. (1985)^[22]^ | No appropriate comparison data. |
| Formigli *et al.* (1990)^[23]^ | No appropriate comparison data. |
| Friedman (1977)^[24]^ | No appropriate comparison data. |
| Ghuman *et al.* (2016)^[25]^ | No appropriate comparison data. |
| Gibbons et al. (2011)^[26]^ | No appropriate comparison data. |
| Glezerman and Potashnik (1981)^[27]^ | No appropriate comparison data. |
| Goss (1975)^[28]^ | No appropriate comparison data. |
| Guerif *et al.* (2002)^[29]^ | No appropriate comparisons. |
| Guerif *et al.* (2004)^[30]^ | No appropriate comparisons. |
| Gunby *et al.* (2005)^[31]^ | No appropriate comparison data. |
| Gunby *et al.* (2006)^[32]^ | No appropriate comparison data. |
| Gunby *et al.* (2007)^[33]^ | No appropriate comparison data. |
| Gunby *et al.* (2008)^[34]^ | No appropriate comparison data. |
| Gunby *et al.* (2009)^[35]^ | No appropriate comparison data. |
| Gunby *et al.* (2010)^[36]^ | No appropriate comparison data. |
| Gunby *et al.* (2011)^[37]^ | No appropriate comparison data. |
| Hedges and Saunders (1993)^[38]^ | No donor sperm outcomes. |
| Horne et al. (1998)^[39]^ | No appropriate comparison data. |
| James et al. (2024)^[40]^ | Conference report, no data. |
| Katzorke et al. (1981)^[41]^ | No appropriate comparison data. |
| Lambert (2003)^[42]^ | Review. |
| Lansac and Le Lannou (1994)^[43]^ | No appropriate comparison data. |
| Lansac and Royere (2001)^[44]^ | Review. |
| Liu et al. (2011)^[45]^ | No appropriate comparison data. |
| Lou et al. (2023)^[46]^ | No appropriate comparison data. |
| Luke (2017)^[47]^ | Review. |
| Mochimaru (1979)^[48]^ | No appropriate comparison data. |
| Need et al. (1983)^[49]^ | No appropriate comparison data. |
| Nyboe Andersen *et al.* (2009)^[50]^ | No appropriate comparison data. |
| Oehninger *et al.* (1998)^[51]^ | No appropriate comparison data. |
| Ping *et al.* (2011)^[52]^ | No appropriate comparison data. |
| Ping *et al.* (2011b)^[53]^ | No appropriate comparison data. |
| Porreco and Heyborne (2018)^[54]^ | Review. |
| Fritz *et al.* (2017)^[55]^ | Conference report, no data. |
| Robertson and Sharkey (2016)^[56]^ | No appropriate comparison data. |
| Schoysman and Schoysman-Deboeck (1976)^[57]^ | No appropriate comparison data. |
| Sanger et al. (1979)^[58]^ | No appropriate comparison data. |
| Society for Assisted Reproductive Technology and ASRM (1995)^[59]^ | No donor sperm outcomes. |
| Society for Assisted Reproductive Technology and ASRM (1998)^[60]^ | No donor sperm outcomes. |
| Society for Assisted Reproductive Technology and ASRM (1999)^[61]^ | No donor sperm outcomes. |
| Society for Assisted Reproductive Technology and ASRM (2000)^[62]^ | No donor sperm outcomes. |
| Society for Assisted Reproductive Technology and ASRM (2001)^[63]^ | No donor sperm outcomes. |
| Smith *et al.* (1981)^[64]^ | No appropriate comparison data. |
| Strickler *et al.* (1975)^[65]^ | No appropriate comparison data. |
| Sulewski *et al.* (1978)^[66]^ | No appropriate comparison data. |
| Sun *et al.* (2013)^[67]^ | No appropriate comparison data. |
| Thepot et al. (1996)^[68]^ | Duplicate reference appearing in another format. |
| Thepot and Fdn Francaise (1998)^[69]^ | No appropriate comparison data. |
| Thomopoulos et al. (2013)^[70]^ | Review. |
| Thomopoulos et al. (2017)^[71]^ | No appropriate comparison data. |
| Trounson et al. (1981)^[72]^ | No appropriate comparison data. |
| Virro and Shewchuk (1984)^[73]^ | No appropriate comparison data. |
| Wang et al. (2002)^[74]^ | No appropriate comparison data. |
| Yeh and Seibel (1987)^[75]^ | No appropriate comparison data. |
| Zhang et al. (2017)^[76]^ | No appropriate comparison data. |
| Zhang et al. (2019)^[77]^ | No appropriate comparison data. |

**References**

1. Adesiyun AG. Referral for assisted reproductive technology: Indications and treatment outcome. Annals of African Medicine 2011; 10(4): 316-8.

2. Alfredsson J. Incidence of spontaneous abortion following artificial insemination by donor. International journal of fertility 1988; 33(4): 241-5.

3. Allen VM, Wilson RD, Cheung A, Genetics C, Reproductive E, Infertility C. RETIRED: Pregnancy outcomes after assisted reproductive technology. Journal of obstetrics and gynaecology Canada : JOGC = Journal d'obstetrique et gynecologie du Canada : JOGC 2006; 28(3): 220-33.

4. Assisted reproductive technology in the United States and Canada: 1992 results generated from The American Fertility Society/Society for Assisted Reproductive Technology Registry. Fertility and Sterility 1994; 62(6 I): 1121-8.

5. Amuzu B, Laxova R, Shapiro SS. PREGNANCY OUTCOME, HEALTH OF CHILDREN, AND FAMILY ADJUSTMENT AFTER DONOR INSEMINATION. Obstetrics and Gynecology 1990; 75(6): 899-905.

6. Amuzu BJ, Shapiro SS. Variation in spontaneous abortion rate relates to the indication for therapeutic donor insemination. Obstetrics and Gynecology 1993; 82(1): 128-31.

7. Andersen AN, Goossens V, Ferraretti AP, et al. Assisted reproductive technology in Europe, 2004: results generated from European registers by ESHRE. Human Reproduction 2008; 23(4): 756-71.

8. Arcos-Machancoses J, Marin Reina P, Martinez F, Jimenez Busselo M, Perez-Aytes A. Silver-Rusell syndrome caused by epigenetic alteration in a child conceived by intrauterine insemination from donor sperm. American Journal of Medical Genetics Part A 2015; 167(11): 2861-4.

9. Botchan A, Hauser R, Gamzu R, Yogev L, Paz G, Yavetz H. Results of 6139 artificial insemination cycles with donor spermatozoa. Human Reproduction 2001; 16(11): 2298-304.

10. Bromwich P, Kilpatrick M, Newton JR. ARTIFICIAL-INSEMINATION WITH FROZEN STORED DONOR SEMEN. British Journal of Obstetrics and Gynaecology 1978; 85(9): 641-4.

11. Bustillo M, Zarutskie P. Assisted reproductive technology in the United States and Canada: 1994 results generated from the American Society for Reproductive Medicine/Society for Assisted Reproductive Technology Registry. Fertility and Sterility 1996; 66(5): 697-705.

12. Society for Assisted Reproductive TASfR, Medicine. Assisted reproductive technology in the United States: 1996 Results generated from the American Society for Reproductive Medicine/Society for Assisted Reproductive Technology Registry. Fertility and Sterility 1999; 71(5): 798-807.

13. Castillo CM, Horne G, Fitzgerald CT, Johnstone ED, Brison DR, Roberts SA. The impact of IVF on birthweight from 1991 to 2015: a cross-sectional study. Human reproduction (Oxford, England) 2019; 34(5): 920-31.

14. Clarke GN, Bourne H, Hill P, et al. Artificial insemination and in-vitro fertilization using donor spermatozoa: A report on 15 years of experience. Human Reproduction 1997; 12(4): 722-6.

15. Cottinet D, Gagnaire JC, Lansac J, Thirion A. Use of frozen sperm in artificial insemination with donor. GYNECOLOGIE 1974; 25(6): 571-5.

16. de Mouzon J, Goossens V, Bhattacharya S, et al. Assisted reproductive technology in Europe, 2006: results generated from European registers by ESHRE. Human Reproduction 2010; 25(8): 1851-62.

17. Dixon RE, Buttram Jr VC. Artificial insemination using donor semen: a review of 171 cases. Fertility and Sterility 1976; 27(2): 130-4.

18. Dyer SJ, Kruger TF. Assisted reproductive technology in South Africa: First results generated from the South African Register of Assisted Reproductive Techniques. Samj South African Medical Journal 2012; 102(3): 167-70.

19. Esteves SC. Long-term outcomes of elective human sperm cryostorage: Editorial comment. International Braz J Urol 2002; 28(2): 168-9.

20. Fang Y-Y, Wu Q-J, Zhang T-N, et al. Assessment of the development of assisted reproductive technology in Liaoning province of China, from 2012 to 2016. Bmc Health Services Research 2018; 18.

21. Ferrara I, Balet R, Grudzinskas JG. Intrauterine donor insemination in single women and lesbian couples: a comparative study of pregnancy rates. Human Reproduction 2000; 15(3): 621-5.

22. Formigli L, Formigli G, Gottardi L. Artificial insemination by donor results in relation to husband's semen. Systems Biology in Reproductive Medicine 1985; 14(2-3): 209-11.

23. Formigli L, Coglitore MT, Roccio C, Belotti G, Stangalini A, Formigli G. One-hundred-and-six gamete intra-fallopian transfer procedures with donor semen. Human Reproduction 1990; 5(5): 549-52.

24. Friedman S. Artificial donor insemination with frozen human semen. Fertility and Sterility 1977; 28(11): 1230-3.

25. Ghuman NK, Mair E, Pearce K, Choudhary M. Does age of the sperm donor influence live birth outcome in assisted reproduction? Human Reproduction 2016; 31(3): 582-90.

26. Gibbons WE, Cedars M, Ness RB, Soc Assisted Reprod Technologies W. Toward understanding obstetrical outcome in advanced assisted reproduction: varying sperm, oocyte, and uterine source and diagnosis. Fertility and Sterility 2011; 95(5): 1645-U147.

27. Glezerman M, Potashnik G. Artificial Insemination using Fresh Donor Semen/Künstliche heterologe Befruchtung mit Frischsamen. Andrologia 1988; 20(5): 384-8.

28. Goss DA. Current status of artificial insemination with donor semen. American Journal of Obstetrics and Gynecology 1975; 122(2): 246-52.

29. Guerif F, Fourquet F, Marret H, et al. Cohort follow-up of couples with primary infertility in an ART programme using frozen donor semen. Human Reproduction 2002; 17(6): 1525-31.

30. Guerif F, Saussereau MH, Barthelemy C, et al. Efficacy of IVF using frozen donor semen in cases of previously failed DI cycles compared with tubal infertility: a cohort study. Reproductive Biomedicine Online 2004; 9(4): 404-8.

31. Gunby J, Daya S, Fertili IVFDGC. Assisted reproductive technologies (ART) in Canada: 2001 results from the Canadian ART Register. Fertility and Sterility 2005; 84(3): 590-9.

32. Gunby J, Daya S, Canadian IVFDG. Assisted reproductive technologies (ART) in Canada: 2002 results from the Canadian ART Register. Fertility and Sterility 2006; 86(5): 1356-64.

33. Gunby J, Daya S. Assisted reproductive technologies (ART) in Canada: 2003 results from the Canadian ART Register. Fertility and Sterility 2007; 88(3): 550-9.

34. Gunby J, Bissonnette F, Librach C, Cowan L, Fertil IVFDGC. Assisted reproductive technologies (ART) in Canada: 2004 results from the Canadian ART Register. Fertility and Sterility 2008; 89(5): 1123-32.

35. Gunby J, Bissonnette F, Librach C, Cowan L, Fertili IVFDGC. Assisted reproductive technologies in Canada: 2005 results from the Canadian Assisted Reproductive Technologies Register. Fertility and Sterility 2009; 91(5): 1721-30.

36. Gunby J, Bissonnette F, Librach C, Cowan L, Canadian F, Andrology S. Assisted reproductive technologies (ART) in Canada: 2006 results from the Canadian ART Register. Fertility and Sterility 2010; 93(7): 2189-201.

37. Gunby J, Bissonnette F, Librach C, Cowan L, Canadian IVFDG. Assisted reproductive technologies (ART) in Canada: 2007 results from the Canadian ART Register. Fertility and Sterility 2011; 95(2).

38. Hedges AK, Saunders DM. CLINICAL OUTCOMES OF PREGNANCIES ACHIEVED BY MICROINSEMINATION BY SPERM TRANSFER. Fertility and Sterility 1993; 60(4): 720-3.

39. Horne G, Jamaludin A, Critchlow JD, et al. A 3 year retrospective review of intrauterine insemination, using cryopreserved donor spermatozoa and cycle monitoring by urinary or serum luteinizing hormone measurements. Human Reproduction 1998; 13(11): 3045-8.

40. James KE, Hsu S, Souter I, Bormann C, Powe CE. 484 Donor gamete status and hypertensive disorders of pregnancy in pregnancies conceived with assisted reproductive technologies. American Journal of Obstetrics and Gynecology 2024; 230(1): S265.

41. Katzorke T, Propping D, Tauber PF. Results of donor artificial insemination (AID) in 415 couples. International journal of fertility 1981; 26(4): 260-6.

42. Lambert RD. Safety issues in assisted reproductive technology: Aetiology of health problems in singleton ART babies. Human Reproduction 2003; 18(10): 1987-91.

43. Lansac J, Le Lannou D. Reproductive health care policies around the world: Sperm donation and practice of AID in France. Journal of Assisted Reproduction and Genetics 1994; 11(5): 231-6.

44. Lansac J, Royère D. Follow-up studies of children born after frozen sperm donation. Human Reproduction Update 2001; 7(1): 33-7.

45. Liu W, Li XF, Xi WY, Tan L, Fan LQ, Lu GX. The follow-up study of pregnancy and neonates outcomes of 10648 AID cycles. Human Reproduction 2011; 26: i277-i8.

46. Luo X-F, Wu H-L, Ji X-R, et al. Comparison of Clinical Outcomes, Risks, and Costs for 20,910 Donor In Vitro Fertilization and 16,850 Donor Artificial Insemination Treatment Cycles: A Retrospective Analysis in China. Journal of Clinical Medicine 2023; 12(3).

47. Luke B. Pregnancy and birth outcomes in couples with infertility with and without assisted reproductive technology: with an emphasis on US population-based studies. American Journal of Obstetrics and Gynecology 2017; 217(3): 270-81.

48. Mochimaru F. Artificial insemination with frozen donor semen: Its current status and follow-up studies. The Keio Journal of Medicine 1979; 28(1): 33-48.

49. Need JA, Bell B, Meffin E, Jones WR. Pre-eclampsia in pregnancies from donor inseminations. Journal of reproductive immunology 1983; 5(6): 329-38.

50. Andersen AN, Goossens V, Bhattacharya S, et al. Assisted reproductive technology and intrauterine inseminations in Europe, 2005: results generated from European registers by ESHRE. Human Reproduction 2009; 24(6): 1267-87.

51. Oehninger S, Chaturvedi S, Toner J, et al. Semen quality: Is there a paternal effect on pregnancy outcome in in-vitro fertilization/intracytoplasmic sperm injection? Human Reproduction 1998; 13(8): 2161-4.

52. Ping P, Cao X, Yan F, et al. Retrospective study of sperm donation and its clinical use of Shanghai Human Sperm Bank. Chinese Journal of Andrology 2011; 25(10): 47-50.

53. Ping P, Zhu W-B, Zhang X-Z, et al. Sperm donation and its application in China: a 7-year multicenter retrospective study. Asian Journal of Andrology 2011b; 13(4): 644-8.

54. Porreco RP, Heyborne KD. Immunogenesis of preeclampsia: lessons from donor gametes. Journal of Maternal-Fetal & Neonatal Medicine 2018; 31(9): 1220-6.

55. Fritz R, Jindal SK, Yu B, Vega MG, Buyuk E. DOES DONOR SPERM AFFECT BIRTH WEIGHT (BW), PRETERM BIRTH (PB), AND MISCARRIAGE RATES IN FRESH AUTOLOGOUS IN VITRO FERTILIZATION (IVF) CYCLES? ANALYSIS OF 46,061 CYCLES REPORTED TO SART. Fertility and Sterility 2017; 107(3): E30-E.

56. Robertson SA, Sharkey DJ. Seminal fluid and fertility in women. Fertility and Sterility 2016; 106(3): 511-9.

57. Schoysman R, Schoysman-Deboeck A. Artificial insemination with donor sperm: clinical results in 583 pregnancies. Contraception Fertilite Sexualite 1976; 4(7): 591-6.

58. Sanger WG, Schwartz MB, Housel G. The use of frozen-thawed for therapeutic insemination (donor). International journal of fertility 1979; 24(4): 267-9.

59. Society For Assisted Reproductive T, American Society F, Reproductive M. Assisted reproductive technology in the United States and Canada: 1993 results generated from the American Society for Reproductive Medicine/Society for Assisted Reproductive Technology Registry. Fertility and Sterility 1995; 64(1): 13-21.

60. Society For Assisted Reproductive T, American Society F, Reproductive M. Assisted reproductive technology in the United States and Canada: 1995 results generated from the American Society for Reproductive Medicine/Society for Assisted Reproductive Technology Registry. Fertility and Sterility 1998; 69(3): 389-98.

61. Amer Soc Reprod M, Amer Soc Reprod M, Soc Assisted Reprod T. Assisted reproductive technology in the United States: 1999 results generated from the American Society for Reproductive Medicine/Society for Assisted Reproductive Technology Registry. Fertility and Sterility 2002; 78(5): 918-31.

62. Society Assisted Reproductive T, American Society Reproductive M. Assisted reproductive technology in the United States: 2000 results generated from the American Society for Reproductive Medicine/Society for Assisted Reproductive Technology Registry. Fertility and Sterility 2004; 81(5): 1207-20.

63. Society Assisted Reproductive T, American Society Reproductive M. Assisted reproductive technology in the United States: 2001 results generated from the American Society for Reproductive Medicine/Society for Assisted Reproductive Technology registry. Fertility and Sterility 2007; 87(6): 1253-66.

64. Smith KD, Rodriguez-Rigau LJ, Steinberger E. The influence of ovulatory dysfunction and timing of insemination on the success of artificial insemination donor (AID) with fresh or cryopreserved semen. Fertility and Sterility 1981; 36(4): 496-502.

65. Strickler RC, Keller DW, Warren JC. Artificial Insemination with Fresh Donor Semen. New England Journal of Medicine 1975; 293(17): 848-53.

66. Sulewski JM, Eisenberg F, Stenger VG. A longitudinal analysis of artificial insemination with donor semen. Fertility and Sterility 1978; 29(5): 527-31.

67. Sun H-Y, Gu M-J, Sun Y-Y, et al. Analysis of the pregnancy outcomes of 13 723 tubes of sperm specimens from the sperm bank. Zhonghua nan ke xue = National journal of andrology 2013; 19(9): 798-801.

68. Thepot F, Mayaux MJ, Czyglick F, Wack T, Selva J, Jalbert P. Incidence of birth defects after artificial insemination with frozen donor spermatozoa: A collaborative study of the French CECOS Federation on 11535 pregnancies. Human Reproduction 1996; 11(10): 2319-23.

69. Thepot F, Fdn Francaise C. Result 1997 of gamete cryopreservation and gamete donation in French. Contraception Fertilite Sexualite 1998; 26(7-8): 476-80.

70. Thomopoulos C, Tsioufis C, Michalopoulou H, Makris T, Papademetriou V, Stefanadis C. Assisted reproductive technology and pregnancy-related hypertensive complications: a systematic review. Journal of Human Hypertension 2013; 27(3): 148-57.

71. Thomopoulos C, Salamalekis G, Kintis K, et al. Risk of hypertensive disorders in pregnancy following assisted reproductive technology: overview and meta-analysis. Journal of Clinical Hypertension 2017; 19(2): 173-83.

72. Trounson AO, Matthews CD, Kovacs GT, et al. Artificial insemination by frozen donor semen: results of multicentre Australian experience. International Journal of Andrology 1981; 4(1-6): 227-34.

73. Virro MR, Shewchuk AB. Pregnancy outcome in 242 conceptions after artificial insemination with donor sperm and effects of maternal age on the prognosis for successful pregnancy. American Journal of Obstetrics and Gynecology 1984; 148(5): 518-24.

74. Wang JX, Norman RJ, Kristiansson P. The effect of various infertility treatments on the risk of preterm birth. Human Reproduction 2002; 17(4): 945-9.

75. Yeh J, Seibel MM. Artificial insemination with donor sperm: A review of 108 patients. Obstetrics and Gynecology 1987; 70(3 PART I): 313-6.

76. Zhang Q, Song G, Zhong X, et al. Analysis of the clinical outcome of 10 690 artificial insemination cycles with donor sperma. Chinese Journal of Reproduction and Contraception 2017; 37(2): 101-5.

77. Zhang A, Ma X, Zhang L, Zhang X, Wang W. Pregnancy and offspring outcomes after artificial insemination with donor sperm A retrospective analysis of 1805 treatment cycles performed in Northwest China. Medicine 2019; 98(16).

**Table S2. The results of quality assessment using the Joanna Briggs Institute (JBI) critical appraisal tools for cohort studies.**

| **Study (year)** | **Q1** | **Q2** | **Q3** | **Q4** | **Q5** | **Q6** | **Q7** | **Q8** | **Q9** | **Q10** | **Q11** | **Total** | **Quality** |
| --- | --- | --- | --- | --- | --- | --- | --- | --- | --- | --- | --- | --- | --- |
| Adams et al. 2017 | N | Y | Y | Y | Y | Y | Y | Y | U | N | Y | 77.27% | High |
| Allen et al. 2022 | Y | Y | Y | Y | Y | Y | Y | Y | U | N | Y | 86.36% | High |
| Allen et al. 2023 | Y | Y | Y | Y | Y | Y | Y | Y | Y | N | Y | 90.91% | High |
| Alorf et al. 2024 | Y | Y | Y | Y | Y | Y | Y | Y | U | N | Y | 86.36% | High |
| Azem et al. 1994 | Y | Y | Y | N | N | Y | Y | Y | U | N | N | 59.09% | Medium |
| Bai et al. 2020 | Y | Y | Y | N | N | Y | Y | Y | Y | N | N | 63.64% | Medium |
| Bortoletto et al. 2020 | Y | Y | Y | Y | Y | Y | Y | Y | U | N | Y | 86.36% | High |
| Bu et al. (2016) | Y | Y | Y | Y | Y | Y | Y | Y | U | N | Y | 86.36% | High |
| Chen et al. 2018 | Y | Y | Y | N | N | Y | Y | Y | Y | Y | N | 72.73% | High |
| Cheng et al. 2018 | Y | Y | Y | N | N | Y | Y | Y | U | N | N | 59.09% | Medium |
| Davies et al. (2012) | N | Y | Y | Y | Y | Y | Y | Y | Y | N | Y | 81.82% | High |
| Dong et al. 2011 | Y | Y | Y | N | N | Y | Y | Y | U | N | N | 59.09% | Medium |
| Dunietz et al. 2017 | Y | Y | Y | Y | Y | Y | Y | Y | N | N | Y | 81.82% | High |
| de Mouzon et al. 2007 | Y | Y | Y | N | N | Y | Y | Y | U | N | N | 59.09% | Medium |
| Esteves et al. 2014 | Y | Y | Y | N | N | Y | Y | Y | U | N | N | 59.09% | Medium |
| Frank et al.2022 | Y | Y | Y | N | N | Y | Y | Y | U | N | N | 59.09% | Medium |
| Gao et al. 2022 | Y | Y | Y | Y | Y | Y | Y | Y | Y | N | Y | 90.91% | High |
| Gaudoin et al. 2003 | N | Y | Y | N | N | Y | Y | Y | U | N | N | 50.00% | Medium |
| Gerkowicz et al. 2018 | Y | Y | Y | Y | Y | Y | Y | Y | Y | N | Y | 90.91% | High |
| Guo et al. 2017 | Y | Y | Y | N | N | Y | Y | Y | U | N | N | 59.09% | Medium |
| Hall et al. 2001 | N | Y | Y | N | N | Y | Y | Y | Y | N | N | 54.55% | Medium |
| Han et al. 2010 | Y | Y | Y | N | N | Y | Y | Y | U | N | N | 59.09% | Medium |
| Hinduja et al. 2008 | Y | Y | Y | N | N | Y | Y | Y | U | N | N | 59.09% | Medium |
| Hoy et al. 1999 | N | Y | Y | Y | Y | Y | Y | Y | Y | N | Y | 81.82% | High |
| Huang et al. 2016 | N | Y | Y | N | N | Y | Y | Y | U | N | N | 50.00% | Medium |
| Kamath et al. 2018 | Y | Y | Y | Y | Y | Y | Y | Y | N | N | Y | 81.82% | High |
| Kennedy et al. 2019 | Y | Y | Y | Y | Y | Y | Y | Y | Y | N | Y | 90.91% | High |
| Kyrou et al. 2010 | Y | Y | Y | N | N | Y | Y | Y | N | N | N | 54.55% | Medium |
| Laivuori et al. 1998 | N | Y | Y | N | N | Y | Y | Y | N | N | N | 45.45% | Medium |
| Lansac et al. 1997 | Y | Y | Y | N | N | Y | Y | Y | U | N | N | 59.09% | Medium |
| Liu et al. 2017 | Y | Y | Y | N | N | Y | Y | Y | U | N | N | 59.09% | Medium |
| Luke et al. 2016 | Y | Y | Y | Y | Y | Y | Y | Y | Y | N | Y | 90.91% | High |

**Table S2. (Contituned).**

| **Study (year)** | **Q1** | **Q2** | **Q3** | **Q4** | **Q5** | **Q6** | **Q7** | **Q8** | **Q9** | **Q10** | **Q11** | **Total** | **Quality** |
| --- | --- | --- | --- | --- | --- | --- | --- | --- | --- | --- | --- | --- | --- |
| Malchau et al. 2014 | Y | Y | Y | Y | Y | Y | Y | Y | U | N | Y | 86.36% | High |
| Ni et al. 2022 | Y | Y | Y | N | N | Y | Y | Y | U | N | Y | 68.18% | Medium |
| Oehninger 1998 | Y | Y | Y | N | N | Y | Y | Y | U | N | Y | 68.18% | Medium |
| Plasencia et al. 2004 | Y | Y | Y | N | N | Y | Y | Y | U | N | Y | 68.18% | Medium |
| Prados et al. 2017 | Y | Y | Y | N | N | Y | Y | Y | U | N | N | 59.09% | Medium |
| Robinson et al. 1993 | Y | Y | Y | N | N | Y | Y | Y | U | N | N | 59.09% | Medium |
| Ruiter-Ligeti et al. 2020 | Y | Y | Y | N | N | Y | Y | Y | U | N | Y | 68.18% | Medium |
| Saavedra-Saavedra et al. 2012 | Y | Y | Y | N | N | Y | Y | Y | U | N | Y | 68.18% | Medium |
| Salha et al. 1999 | Y | Y | Y | N | N | Y | Y | Y | U | N | N | 59.09% | Medium |
| Scarselli et al. 2018 | Y | Y | Y | N | N | Y | Y | Y | U | N | N | 59.09% | Medium |
| Smith et al.1997 | Y | Y | Y | N | N | Y | Y | Y | U | N | Y | 68.18% | Medium |
| Su et al. 2014 | Y | Y | Y | N | N | Y | Y | Y | U | N | N | 59.09% | Medium |
| Sun et al. 2022 | Y | Y | Y | N | N | Y | Y | Y | U | N | Y | 68.18% | Medium |
| Thapar et al. 2007 | Y | Y | Y | N | N | Y | Y | Y | U | N | N | 59.09% | Medium |
| Varma et al. 1987 | N | Y | Y | N | N | Y | Y | Y | Y | N | N | 54.55% | Medium |
| Verp et al. 1983 | N | Y | Y | N | N | Y | Y | Y | Y | N | N | 54.55% | Medium |
| Wang et al. 2019 | Y | Y | Y | N | N | Y | Y | Y | U | N | Y | 68.18% | Medium |
| Warnes et al. 1998 | Y | Y | Y | N | N | Y | Y | Y | U | N | N | 59.09% | Medium |
| Xu et al. 2014 | Y | Y | Y | N | N | Y | Y | Y | U | N | Y | 68.18% | Medium |
| Yan et al. 2011 | Y | Y | Y | N | N | Y | Y | Y | U | N | N | 59.09% | Medium |
| Yan et al. 2015 | Y | Y | Y | N | N | Y | Y | Y | U | N | N | 59.09% | Medium |
| Yang et al. 2021 | Y | Y | Y | N | N | Y | Y | Y | U | N | N | 59.09% | Medium |
| Yovich et al. 1988 | Y | Y | Y | N | N | Y | Y | Y | U | N | N | 59.09% | Medium |
| Yu et al. 2018 | Y | Y | Y | Y | Y | Y | Y | Y | U | N | Y | 86.36% | High |
| Yu et al. 2018b | Y | Y | Y | N | N | Y | Y | Y | U | N | N | 59.09% | Medium |
| Zamora et al. 2014 | Y | Y | Y | N | N | Y | Y | Y | N | N | Y | 63.64% | Medium |
| Zhang et al. 2014 | Y | Y | Y | N | N | Y | Y | Y | U | N | Y | 68.18% | Medium |
| Zheng et al. 2016 | Y | Y | Y | N | N | Y | Y | Y | U | N | N | 59.09% | Medium |
| Zhou et al. 2018 | Y | Y | Y | N | N | Y | Y | Y | U | N | N | 59.09% | Medium |
| Zhu et al. 2021 | Y | Y | Y | N | N | Y | Y | Y | U | N | N | 59.09% | Medium |
| Zhu et al. 2022 | Y | Y | Y | N | N | Y | Y | Y | U | N | N | 59.09% | Medium |
| Zhu et al. 2024 | Y | Y | Y | N | N | Y | Y | Y | U | N | Y | 68.18% | Medium |

Note：Q, question; Y, yes; N, no; U, unclear; NA, not applicable.

Q1: Were the two groups similar and recruited from the same population?

Q2: Were the exposures measured similarly to assign people to both exposed and unexposed groups?

Q3: Was the exposure measured in a valid and reliable way?

Q4: Were confounding factors identified?

Q5: Were strategies to deal with confounding factors stated?

Q6: Were the groups/participants free of the outcome at the start of the study (or at the moment of exposure)?

Q7: Were the outcomes measured in a valid and reliable way?

Q8: Was the follow up time reported and sufficient to be long enough for outcomes to occur?

Q9: Was follow up complete, and if not, were the reasons to loss to follow up described and explored?

Q10: Were strategies to address incomplete follow up utilized?

Q11: Was appropriate statistical analysis used?

**Table S3.** GRADE assessment of the cumulative evidence for the association between different sperm sources and pregnancy outcomes, obstetric outcomes, and perinatal outcomes.

| Pregnancy outcomes | Number of studies | Pooled effect estimates | Quality assessment | | | | | Quality of evidence |
| --- | --- | --- | --- | --- | --- | --- | --- | --- |
|  |  |  | Risk of bias^a^ | Inconsistency^b^ | Indirectness^c^ | Imprecision^d^ | Publication bias ^e^ |  |
| Clinical pregnancy | 23 | 1.27 (1.08, 1.48) | Not serious | Very serious | Direct | Not serious | Undetected | Very low |
| Ectopic pregnancy | 11 | 0.87(0.67, 1.14) | Not serious | Serious | Direct | Serious | Undetected | Very low |
| Biochemical pregnancy | 7 | 0.85 (0.81, 0.88) | Not serious | Not serious | Direct | Not serious | Undetected | Moderate |
| Miscarriage | 27 | 0.91 (0.84, 1.00) | Not serious | Serious | Direct | Not serious | Undetected | Low |
| Pregnancy-induced hypertension | 14 | 1.19 (1.05, 1.36) | Not serious | Not serious | Direct | Not serious | Undetected | Moderate |
| Pre-eclampsia | 11 | 1.35 (1.06, 1.74) | Not serious | Not serious | Direct | Not serious | Undetected | Moderate |
| Gestational diabetes | 8 | 1.04 (0.71, 1.50) | Not serious | Serious | Direct | Serious | Undetected | Very low |
| Placenta praevia | 3 | 1.00 (0.58, 1.71) | Not serious | Not serious | Direct | Serious | Undetected | Low |
| Preterm birth | 23 | 0.97 (0.92, 1.02) | Not serious | Not serious | Direct | Serious | Undetected | Low |
| Very preterm birth | 8 | 0.88 (0.80, 0.96) | Not serious | Not serious | Direct | Not serious | Undetected | Moderate |
| Low birthweight | 25 | 0.96 (0.89, 1.04) | Not serious | Serious | Direct | Serious | Undetected | Very low |
| Very low birthweight | 9 | 0.89 (0.81, 0.98) | Not serious | Not serious | Direct | Not serious | Undetected | Moderate |
| Small for gestational age | 8 | 0.96 (0.78, 1.18) | Not serious | Serious | Direct | Serious | Undetected | Very low |
| High birth weight | 11 | 1.06 (0.94, 1.20) | Not serious | Not serious | Direct | Serious | Undetected | Low |
| Very high birth weight | 3 | 1.15 (1.01, 1.31) | Not serious | Not serious | Direct | Not serious | Undetected | Moderate |
| Large for gestational age | 4 | 1.04 (0.91, 1.18) | Not serious | Not serious | Direct | Serious | Undetected | Very low |
| Still birth | 10 | 1.11 (0.95, 1.31) | Not serious | Not serious | Direct | Serious | Undetected | Low |
| Neonatal death | 3 | 0.77 (0.35, 1.70) | Not serious | Not serious | Direct | Serious | Undetected | Low |
| Perinatal death | 3 | 1.05 (0.75, 1.48) | Not serious | Not serious | Direct | Serious | Undetected | Low |
| Congenital anomaly | 24 | 0.98 (0.86, 1.11) | Not serious | Not serious | Direct | Serious | Undetected | Low |

a. Risk of bias was evaluated based on the inclusion criteria.

b. Inconsistency was evaluated based on I^2^ statistics: I^2^<50%, not serious; 50%≤ I^2^ <80%, serious; I^2^ ≥80%, very serious.

c. Indirectness: we considered no indirectness if the evidence directly came from the studies related to the topic of meta-analysis,.

d. Imprecision was assessed based on the 95% CI of β.

e. Publication bias was assessed based on Egger’s regression test for asymmetry.

**Table S4. Results of univariable regression analysis.**

| **Outcomes** | **Moderator** | **QM** | ***P* value** | **R² (%)** |
| --- | --- | --- | --- | --- |
| Clinical pregnancy |  |  |  |  |
|  | Adjusted confounding factors | 0.027 | 0.869 | 0 |
|  | Model of conception | 16.68 | <0.001 | 51.24 |
|  | Location | 0.009 | 0.923 | 0 |
| Ectopic pregnancy |  |  |  |  |
|  | Adjusted confounding factors | 0.719 | 0.396 | 0 |
|  | Model of conception | 5.457 | 0.065 | 58.56 |
|  | Location | 1.084 | 0.298 | 19.41 |
| Miscarriage |  |  |  |  |
|  | Adjusted confounding factors | 1.108 | 0.292 | 0.27 |
|  | Model of conception | 7.795 | 0.020 | 11.31 |
|  | Location | 0.125 | 0.724 | 0.00 |
| Gestational diabetes |  |  |  |  |
|  | Adjusted confounding factors | 0.875 | 0.350 | 0 |
|  | Model of conception | 0.985 | 0.611 | 0 |
|  | Location | 5.498 | 0.064 | 60.81 |
| Low birthweight |  |  |  |  |
|  | Adjusted confounding factors | 0.101 | 0.751 | 0.00 |
|  | Model of conception | 17.623 | 0.002 | 76.56 |
|  | Location | 0.119 | 0.730 | 0.00 |
| Samll for gestational age |  |  |  |  |
|  | Adjusted confounding factors | 1.381 | 0.240 | 6.86 |
|  | Model of conception | 3.744 | 0.291 | 13.50 |
|  | Location | 1.550 | 0.213 | 12.17 |

**Table S5. Results of effect weighting analysis.**

| **Study** | **Outcomes** | **Pooled RR (after weighting)** | **Lower**  **95% CI** | **Upper 95% CI** | **Difference from original pooled RR** |
| --- | --- | --- | --- | --- | --- |
| Allen et al. 2023 | Biochemical pregnancy | 0.942 | 0.761 | 1.123 | 0.098 |
| Bortoletto et al. 2020 | Biochemical pregnancy | 0.841 | 0.806 | 0.877 | -0.002 |
| Gao et al. 2022 | Biochemical pregnancy | 0.842 | 0.806 | 0.878 | -0.001 |
| Scarselli et al. 2018 | Biochemical pregnancy | 0.844 | 0.808 | 0.879 | 0.000 |
| Sun et al. 2022 | Biochemical pregnancy | 0.843 | 0.807 | 0.878 | -0.001 |
| Yovich et al. 1988 | Biochemical pregnancy | 0.844 | 0.809 | 0.879 | 0.000 |
| Zhu et al. 2022 | Biochemical pregnancy | 0.844 | 0.808 | 0.879 | 0.000 |
| Allen et al. 2022 (singletons) | Birth defect | 0.961 | 0.840 | 1.083 | 0.077 |
| Allen et al. 2022 (twins) | Birth defect | 0.875 | 0.772 | 0.979 | -0.009 |
| Bai et al. 2020 | Birth defect | 0.884 | 0.782 | 0.987 | 0.000 |
| Chen et al. 2018 | Birth defect | 0.885 | 0.788 | 0.982 | 0.001 |
| Davies et al. 2012 | Birth defect | 0.859 | 0.759 | 0.959 | -0.025 |
| Esteves et al. 2014 | Birth defect | 0.884 | 0.787 | 0.981 | 0.000 |
| Guo et al. 2017 | Birth defect | 0.884 | 0.786 | 0.981 | 0.000 |
| Han et al. 2010 | Birth defect | 0.886 | 0.788 | 0.984 | 0.002 |
| Hoy et al. 1999 | Birth defect | 0.860 | 0.758 | 0.963 | -0.024 |
| Huang et al. 2016 | Birth defect | 0.883 | 0.786 | 0.981 | -0.001 |
| Lansac et al. 1997 | Birth defect | 0.898 | 0.794 | 1.003 | 0.014 |
| Liu et al. 2017 | Birth defect | 0.883 | 0.786 | 0.981 | -0.001 |
| Luke et al. 2016 | Birth defect | 0.886 | 0.788 | 0.984 | 0.002 |
| Varma and Patel 1987 | Birth defect | 0.884 | 0.787 | 0.982 | 0.000 |
| Verp et al. 1983 | Birth defect | 0.885 | 0.787 | 0.982 | 0.001 |
| Xu et al. 2014 | Birth defect | 0.886 | 0.788 | 0.983 | 0.002 |
| Yan et al. 2011 | Birth defect | 0.882 | 0.784 | 0.980 | -0.002 |
| Yan et al. 2015 | Birth defect | 0.884 | 0.786 | 0.981 | 0.000 |
| Yang et al. 2021 | Birth defect | 0.884 | 0.786 | 0.981 | 0.000 |
| Yu et al. 2018b | Birth defect | 0.884 | 0.787 | 0.981 | 0.000 |
| Zhou et al. 2018 | Birth defect | 0.880 | 0.783 | 0.978 | -0.004 |
| Zhu et al. 2022 | Birth defect | 0.885 | 0.788 | 0.983 | 0.001 |
| Zhu et al. 2024 | Birth defect | 0.881 | 0.783 | 0.979 | -0.003 |
| Wang et al. 2019 | Birth defect | 0.875 | 0.774 | 0.976 | -0.009 |
| Alorf et al. 2024 | Clinical pregnancy | 1.266 | 1.062 | 1.471 | -0.008 |
| Azem et al. 1994 | Clinical pregnancy | 1.292 | 1.084 | 1.501 | 0.018 |
| Bai et al. 2020 | Clinical pregnancy | 1.228 | 1.087 | 1.370 | -0.046 |
| Bortoletto et al. 2020 | Clinical pregnancy | 1.284 | 1.071 | 1.496 | 0.009 |
| Cheng et al. 2018 | Clinical pregnancy | 1.300 | 1.091 | 1.510 | 0.026 |
| Dong et al. 2011 | Clinical pregnancy | 1.204 | 1.000 | 1.409 | -0.070 |
| Esteves et al. 2014 | Clinical pregnancy | 1.270 | 1.061 | 1.480 | -0.004 |
| Frank et al.2022 | Clinical pregnancy | 1.271 | 1.064 | 1.479 | -0.003 |
| Gao et al. 2022 | Clinical pregnancy | 1.288 | 1.063 | 1.512 | 0.013 |
| Gerkowicz et al. 2018 | Clinical pregnancy | 1.289 | 1.067 | 1.512 | 0.015 |
| Ni et al. 2022 | Clinical pregnancy | 1.292 | 1.079 | 1.504 | 0.017 |
| Oehninger 1998 | Clinical pregnancy | 1.282 | 1.073 | 1.491 | 0.007 |
| Prados et al. 2017 | Clinical pregnancy | 1.289 | 1.074 | 1.504 | 0.015 |
| Ruiter-Ligeti et al. 2020 | Clinical pregnancy | 1.274 | 1.070 | 1.479 | 0.000 |
| Scarselli et al. 2018 | Clinical pregnancy | 1.264 | 1.057 | 1.471 | -0.010 |
| Su et al. 2014 | Clinical pregnancy | 1.285 | 1.075 | 1.495 | 0.010 |
| Sun et al. 2022 | Clinical pregnancy | 1.283 | 1.074 | 1.492 | 0.009 |
| Xu et al. 2014 | Clinical pregnancy | 1.269 | 1.062 | 1.476 | -0.005 |
| Zhang et al. 2014 | Clinical pregnancy | 1.267 | 1.059 | 1.475 | -0.007 |
| Zheng et al. 2016 | Clinical pregnancy | 1.288 | 1.078 | 1.498 | 0.014 |
| Zhou et al. 2018 | Clinical pregnancy | 1.238 | 1.029 | 1.447 | -0.036 |
| Zhu et al. 2021 | Clinical pregnancy | 1.294 | 1.085 | 1.504 | 0.020 |
| Zhu et al. 2022 | Clinical pregnancy | 1.284 | 1.070 | 1.498 | 0.010 |
| Allen et al. 2023 | Ectopic pregnancy | 0.737 | 0.520 | 0.954 | -0.005 |
| Bortoletto et al. 2020 | Ectopic pregnancy | 0.740 | 0.578 | 0.903 | -0.001 |
| Bu et al. 2016 | Ectopic pregnancy | 0.732 | 0.613 | 0.850 | -0.010 |
| Chen et al. 2018 | Ectopic pregnancy | 0.749 | 0.585 | 0.913 | 0.007 |
| de Mouzon et al. 2007 | Ectopic pregnancy | 0.783 | 0.617 | 0.948 | 0.042 |
| Gao et al. 2022 | Ectopic pregnancy | 0.755 | 0.589 | 0.922 | 0.014 |
| Prados et al. 2017 | Ectopic pregnancy | 0.717 | 0.543 | 0.892 | -0.024 |
| Varma and Patel 1987 | Ectopic pregnancy | 0.739 | 0.580 | 0.899 | -0.002 |
| Warnes et al. 1998 | Ectopic pregnancy | 0.753 | 0.557 | 0.950 | 0.012 |
| Yovich et al. 1988 | Ectopic pregnancy | 0.763 | 0.616 | 0.910 | 0.022 |
| Zamora et al. 2014 | Ectopic pregnancy | 0.705 | 0.559 | 0.850 | -0.037 |
| Adams et al. 2017 | Gestational diabetes | 0.913 | 0.603 | 1.223 | -0.005 |
| Chen et al. 2017 | Gestational diabetes | 0.998 | 0.705 | 1.292 | 0.080 |
| Gao et al. 2022 | Gestational diabetes | 0.751 | 0.500 | 1.003 | -0.167 |
| Luke et al. 2016 | Gestational diabetes | 0.927 | 0.583 | 1.271 | 0.008 |
| Thapar et al. 2007 | Gestational diabetes | 0.901 | 0.589 | 1.212 | -0.018 |
| Yan et al. 2015 | Gestational diabetes | 0.993 | 0.709 | 1.276 | 0.074 |
| Chen et al. 2018 | Gestational diabetes | 0.916 | 0.625 | 1.208 | -0.002 |
| Varma and Patel 1987 | Gestational diabetes | 0.914 | 0.647 | 1.180 | -0.005 |
| Allen et al. 2022 (singletons) | High birth weight | 0.908 | 0.681 | 1.134 | -0.065 |
| Allen et al. 2022 (twins) | High birth weight | 0.980 | 0.834 | 1.126 | 0.007 |
| Gao et al. 2022 | High birth weight | 0.993 | 0.841 | 1.145 | 0.021 |
| Huang et al. 2016 | High birth weight | 0.951 | 0.821 | 1.082 | -0.021 |
| Kamath et al. 2018 | High birth weight | 0.909 | 0.684 | 1.134 | -0.063 |
| Liu et al. 2017 | High birth weight | 1.045 | 0.949 | 1.140 | 0.072 |
| Ni et al. 2022 | High birth weight | 0.969 | 0.824 | 1.114 | -0.003 |
| Varma and Patel 1987 | High birth weight | 0.970 | 0.823 | 1.118 | -0.002 |
| Yan et al. 2015 | High birth weight | 0.966 | 0.819 | 1.112 | -0.007 |
| Yang et al. 2021 | High birth weight | 0.994 | 0.845 | 1.142 | 0.021 |
| Zhu et al. 2024 | High birth weight | 0.984 | 0.838 | 1.131 | 0.012 |
| Cheng et al. 2018 | Implantation rate | 1.062 | 0.865 | 1.259 | -0.006 |
| Oehninger 1998 | Implantation rate | 1.033 | 0.832 | 1.234 | -0.035 |
| Scarselli et al. 2018 | Implantation rate | 1.051 | 0.869 | 1.234 | -0.017 |
| Zheng et al. 2016 | Implantation rate | 1.125 | 0.885 | 1.365 | 0.057 |
| Zhu et al. 2021 | Implantation rate | 1.091 | 0.886 | 1.295 | 0.023 |
| Adams et al. 2017 | Large for gestational age | 1.042 | 0.891 | 1.193 | 0.005 |
| Gao et al. 2022 | Large for gestational age | 1.042 | 0.855 | 1.230 | 0.005 |
| Luke et al. 2016 | Large for gestational age | 1.033 | 0.893 | 1.172 | -0.004 |
| Malchau et al. 2014 | Large for gestational age | 1.034 | 0.876 | 1.192 | -0.003 |
| Allen et al. 2023 | Live birth | 1.220 | 1.064 | 1.376 | 0.083 |
| Alorf et al. 2024 | Live birth | 1.136 | 1.077 | 1.196 | -0.001 |
| Bortoletto et al. 2020 | Live birth | 1.132 | 1.072 | 1.193 | -0.005 |
| Esteves et al. 2014 | Live birth | 1.135 | 1.075 | 1.195 | -0.002 |
| Gao et al. 2022 | Live birth | 1.151 | 1.085 | 1.216 | 0.014 |
| Ni et al. 2022 | Live birth | 1.150 | 1.087 | 1.213 | 0.013 |
| Ruiter-Ligeti et al. 2020 | Live birth | 1.137 | 1.077 | 1.197 | 0.000 |
| Scarselli et al. 2018 | Live birth | 1.135 | 1.075 | 1.195 | -0.002 |
| Sun et al. 2022 | Live birth | 1.138 | 1.078 | 1.198 | 0.001 |
| Zhou et al. 2018 | Live birth | 1.038 | 1.004 | 1.072 | -0.099 |
| Zhu et al. 2022 | Live birth | 1.138 | 1.075 | 1.201 | 0.001 |
| Chen et al. 2018 | Live birth | 1.158 | 1.092 | 1.224 | 0.021 |
| Gerkowicz et al. 2018 | Live birth | 1.224 | 1.076 | 1.372 | 0.087 |
| Gao et al. 2022 | Low birth weight | 0.930 | 0.759 | 1.100 | 0.025 |
| Liu et al. 2017 | Low birth weight | 0.884 | 0.732 | 1.036 | -0.021 |
| Thapar et al. 2007 | Low birth weight | 0.875 | 0.713 | 1.037 | -0.030 |
| Yan et al. 2015 | Low birth weight | 0.947 | 0.784 | 1.110 | 0.042 |
| Zhu et al. 2024 | Low birth weight | 0.858 | 0.654 | 1.062 | -0.047 |
| Luke et al. 2016 | Low birth weight | 0.946 | 0.777 | 1.116 | 0.041 |
| Gerkowicz et al. 2018 | Low birth weight | 0.917 | 0.661 | 1.174 | 0.012 |
| Adams et al. 2017 | Low birthweight | 0.912 | 0.832 | 0.992 | -0.010 |
| Allen et al. 2022 (singletons) | Low birthweight | 0.935 | 0.839 | 1.031 | 0.012 |
| Allen et al. 2022 (twins) | Low birthweight | 0.924 | 0.824 | 1.024 | 0.001 |
| Chen et al. 2018 | Low birthweight | 0.933 | 0.858 | 1.008 | 0.011 |
| Gaudoin et al. 2003 | Low birthweight | 0.921 | 0.840 | 1.002 | -0.001 |
| Hoy et al. 1999 | Low birthweight | 0.910 | 0.826 | 0.993 | -0.013 |
| Huang et al. 2016 | Low birthweight | 0.924 | 0.842 | 1.007 | 0.002 |
| Kamath et al. 2018 | Low birthweight | 0.931 | 0.837 | 1.024 | 0.008 |
| Lansac et al. 1997 | Low birthweight | 0.946 | 0.867 | 1.025 | 0.023 |
| Ni et al. 2022 | Low birthweight | 0.924 | 0.842 | 1.005 | 0.001 |
| Varma and Patel 1987 | Low birthweight | 0.921 | 0.840 | 1.003 | -0.001 |
| Yang et al. 2021 | Low birthweight | 0.927 | 0.846 | 1.009 | 0.005 |
| Yu et al. 2018 | Low birthweight | 0.929 | 0.841 | 1.016 | 0.006 |
| Yu et al. 2018b (singletons) | Low birthweight | 0.923 | 0.842 | 1.004 | 0.000 |
| Yu et al. 2018b (twins) | Low birthweight | 0.928 | 0.848 | 1.009 | 0.006 |
| Zhu et al. 2022 | Low birthweight | 0.917 | 0.835 | 0.999 | -0.006 |
| Malchau et al. 2014 (IUI-D vs IUI-H) | Low birthweight | 0.919 | 0.834 | 1.004 | -0.004 |
| Malchau et al. 2014 (IUI-D vs SC) | Low birthweight | 0.892 | 0.821 | 0.963 | -0.030 |
| Allen et al. 2023 | Miscarriage | 0.856 | 0.748 | 0.963 | -0.020 |
| Bortoletto et al. 2020 | Miscarriage | 0.878 | 0.793 | 0.963 | 0.002 |
| Chen et al. 2018 | Miscarriage | 0.873 | 0.790 | 0.956 | -0.003 |
| Cheng et al. 2018 | Miscarriage | 0.875 | 0.793 | 0.957 | -0.001 |
| Esteves et al. 2014 | Miscarriage | 0.874 | 0.791 | 0.957 | -0.001 |
| Hinduja et al. 2008 | Miscarriage | 0.877 | 0.795 | 0.959 | 0.001 |
| Oehninger 1998 | Miscarriage | 0.877 | 0.794 | 0.960 | 0.002 |
| Su et al. 2014 | Miscarriage | 0.875 | 0.792 | 0.957 | -0.001 |
| Varma and Patel 1987 | Miscarriage | 0.872 | 0.793 | 0.952 | -0.003 |
| Warnes et al. 1998 | Miscarriage | 0.882 | 0.800 | 0.963 | 0.006 |
| Xu et al. 2014 | Miscarriage | 0.879 | 0.798 | 0.960 | 0.004 |
| Yan et al. 2015 | Miscarriage | 0.875 | 0.792 | 0.957 | -0.001 |
| Yovich et al. 1988 | Miscarriage | 0.883 | 0.800 | 0.966 | 0.007 |
| Yu et al. 2018 | Miscarriage | 0.853 | 0.758 | 0.948 | -0.022 |
| Zamora et al. 2014 | Miscarriage | 0.846 | 0.764 | 0.928 | -0.030 |
| Zheng et al. 2016 | Miscarriage | 0.876 | 0.793 | 0.958 | 0.000 |
| Zhou et al. 2018 | Miscarriage | 0.907 | 0.831 | 0.983 | 0.032 |
| Ni et al. 2022 | Miscarriage | 0.874 | 0.792 | 0.957 | -0.001 |
| Robinson et al. 1993 | Miscarriage | 0.889 | 0.810 | 0.967 | 0.013 |
| Sun et al. 2022 | Miscarriage | 0.875 | 0.792 | 0.957 | -0.001 |
| Yang et al. 2021 | Miscarriage | 0.880 | 0.795 | 0.965 | 0.005 |
| Gao et al. 2022 (early) | Miscarriage | 0.866 | 0.781 | 0.951 | -0.010 |
| Gao et al. 2022 (late) | Miscarriage | 0.871 | 0.789 | 0.953 | -0.005 |
| de Mouzon et al. 2007 | Miscarriage | 0.901 | 0.821 | 0.981 | 0.025 |
| Plasencia et al. 2004 | Miscarriage | 0.875 | 0.792 | 0.959 | 0.000 |
| Scarselli et al. 2018 | Miscarriage | 0.875 | 0.793 | 0.957 | -0.001 |
| Zhu et al. 2022 | Miscarriage | 0.874 | 0.792 | 0.957 | -0.001 |
| Adams et al. 2017 | Neonatal death | 0.784 | 0.028 | 1.539 | 0.034 |
| Hoy et al. 1999 | Neonatal death | 0.381 | -2.201 | 2.963 | -0.369 |
| Varma and Patel 1987 | Neonatal death | 0.747 | 0.019 | 1.474 | -0.003 |
| Esteves et al. 2014 | Perinatal death | 1.031 | 0.659 | 1.403 | 0.000 |
| Hoy et al. 1999 | Perinatal death | 0.936 | 0.460 | 1.413 | -0.095 |
| Malchau et al. 2014 | Perinatal death | 1.176 | 0.584 | 1.769 | 0.145 |
| Malchau et al. 2014 | Placenta praevia | 0.616 | -0.148 | 1.379 | -0.254 |
| Gao et al. 2022 | Placenta praevia | 1.164 | 0.362 | 1.967 | 0.295 |
| Varma and Patel 1987 | Placenta praevia | 0.862 | 0.302 | 1.422 | -0.007 |
| Adams et al. 2017 | Post-term birth | 1.051 | 1.016 | 1.085 | 0.265 |
| Gao et al. 2022 | Post-term birth | 0.774 | 0.131 | 1.416 | -0.012 |
| Kamath et al. 2018 | Post-term birth | 0.386 | -0.147 | 0.920 | -0.399 |
| Chen et al. 2017 | Pre-eclampsia | 1.081 | 0.526 | 1.637 | 0.000 |
| Gao et al. 2022 | Pre-eclampsia | 1.086 | 0.509 | 1.662 | 0.004 |
| Gaudoin et al. 2003 | Pre-eclampsia | 1.065 | 0.502 | 1.627 | -0.017 |
| Hall et al. 2001 | Pre-eclampsia | 1.092 | 0.514 | 1.671 | 0.011 |
| Hoy et al. 1999 | Pre-eclampsia | 0.992 | 0.433 | 1.551 | -0.089 |
| Kyrou et al. 2010 | Pre-eclampsia | 1.019 | 0.421 | 1.618 | -0.062 |
| Laivuori et al. 1998 | Pre-eclampsia | 1.356 | 1.127 | 1.585 | 0.275 |
| Saavedra-Saavedra et al. 2012 | Pre-eclampsia | 1.054 | 0.473 | 1.635 | -0.027 |
| Salha et al. 1999 | Pre-eclampsia | 1.083 | 0.542 | 1.624 | 0.002 |
| Smith et al.1997 | Pre-eclampsia | 1.060 | 0.522 | 1.598 | -0.021 |
| Yan et al. 2015 | Pre-eclampsia | 1.089 | 0.513 | 1.666 | 0.008 |
| Adams et al. 2017 | Pregnancy induced hypertension | 1.166 | 0.999 | 1.333 | -0.067 |
| Chen et al. 2017 | Pregnancy induced hypertension | 1.233 | 1.082 | 1.384 | -0.001 |
| Chen et al. 2018 | Pregnancy induced hypertension | 1.237 | 1.086 | 1.389 | 0.004 |
| Gao et al. 2022 | Pregnancy induced hypertension | 1.291 | 1.120 | 1.461 | 0.057 |
| Laivuori et al. 1998 | Pregnancy induced hypertension | 1.245 | 1.092 | 1.397 | 0.011 |
| Luke et al. 2016 | Pregnancy induced hypertension | 1.234 | 1.073 | 1.396 | 0.001 |
| Salha et al. 1999 | Pregnancy induced hypertension | 1.233 | 1.082 | 1.384 | 0.000 |
| Smith et al.1997 | Pregnancy induced hypertension | 1.234 | 1.082 | 1.385 | 0.000 |
| Thapar et al. 2007 | Pregnancy induced hypertension | 1.234 | 1.078 | 1.390 | 0.001 |
| Varma and Patel 1987 | Pregnancy induced hypertension | 1.248 | 1.093 | 1.403 | 0.015 |
| Yan et al. 2015 | Pregnancy induced hypertension | 1.239 | 1.086 | 1.392 | 0.006 |
| Hall et al. 2001 | Pregnancy induced hypertension | 1.242 | 1.088 | 1.395 | 0.008 |
| Malchau et al. 2014 | Pregnancy induced hypertension | 1.187 | 1.006 | 1.369 | -0.047 |
| Adams et al. 2017 | Preterm birth | 0.946 | 0.893 | 0.999 | -0.004 |
| Allen et al. 2022 (singletons) | Preterm birth | 0.946 | 0.883 | 1.010 | -0.004 |
| Allen et al. 2022 (twins) | Preterm birth | 0.952 | 0.891 | 1.013 | 0.002 |
| Chen et al. 2017 | Preterm birth | 0.951 | 0.897 | 1.005 | 0.001 |
| Chen et al. 2018 | Preterm birth | 0.953 | 0.900 | 1.007 | 0.003 |
| Dunietz et al. 2017 | Preterm birth | 0.949 | 0.894 | 1.004 | -0.001 |
| Gao et al. 2022 | Preterm birth | 0.952 | 0.896 | 1.008 | 0.001 |
| Gaudoin et al. 2003 | Preterm birth | 0.951 | 0.896 | 1.005 | 0.000 |
| Gerkowicz et al. 2018 | Preterm birth | 0.947 | 0.886 | 1.007 | -0.004 |
| Hoy et al. 1999 | Preterm birth | 0.949 | 0.893 | 1.006 | -0.001 |
| Kamath et al. 2018 | Preterm birth | 0.952 | 0.893 | 1.011 | 0.001 |
| Lansac et al. 1997 | Preterm birth | 0.968 | 0.919 | 1.017 | 0.017 |
| Luke et al. 2016 | Preterm birth | 0.955 | 0.900 | 1.009 | 0.004 |
| Ni et al. 2022 | Preterm birth | 0.950 | 0.896 | 1.005 | 0.000 |
| Varma and Patel 1987 | Preterm birth | 0.951 | 0.897 | 1.005 | 0.001 |
| Yan et al. 2015 | Preterm birth | 0.959 | 0.911 | 1.007 | 0.009 |
| Yang et al. 2021 | Preterm birth | 0.952 | 0.898 | 1.006 | 0.002 |
| Yu et al. 2018 | Preterm birth | 0.951 | 0.895 | 1.006 | 0.000 |
| Yu et al. 2018b | Preterm birth | 0.954 | 0.900 | 1.007 | 0.003 |
| Zhou et al. 2018 | Preterm birth | 0.947 | 0.893 | 1.001 | -0.004 |
| Zhu et al. 2024 | Preterm birth | 0.935 | 0.885 | 0.986 | -0.015 |
| Malchau et al. 2014 (IUI-D vs IUI-H) | Preterm birth | 0.948 | 0.893 | 1.003 | -0.002 |
| Malchau et al. 2014 (IUI-D vs SC) | Preterm birth | 0.941 | 0.892 | 0.991 | -0.009 |
| Adams et al. 2017 | Samll for gestational age | 0.988 | 0.667 | 1.308 | 0.036 |
| Gao et al. 2022 | Samll for gestational age | 1.020 | 0.747 | 1.293 | 0.069 |
| Laivuori et al. 1998 | Samll for gestational age | 0.956 | 0.698 | 1.214 | 0.005 |
| Luke et al. 2016 | Samll for gestational age | 1.007 | 0.729 | 1.284 | 0.055 |
| Varma and Patel 1987 | Samll for gestational age | 0.920 | 0.668 | 1.171 | -0.032 |
| Malchau et al. 2014 (IUI-D vs SC) | Samll for gestational age | 0.829 | 0.673 | 0.985 | -0.123 |
| Malchau et al. 2014 (IUI-D vs IUI-H) | Samll for gestational age | 0.944 | 0.630 | 1.259 | -0.007 |
| Adams et al. 2017 | Still birth | 1.076 | 0.895 | 1.257 | -0.005 |
| Allen et al. 2023 | Still birth | 1.191 | 0.888 | 1.494 | 0.111 |
| Bortoletto et al. 2020 | Still birth | 1.080 | 0.900 | 1.260 | 0.000 |
| Chen et al. 2018 | Still birth | 1.082 | 0.902 | 1.262 | 0.002 |
| Hoy et al. 1999 | Still birth | 1.065 | 0.881 | 1.248 | -0.016 |
| Laivuori et al. 1998 | Still birth | 1.080 | 0.900 | 1.260 | 0.000 |
| Lansac et al. 1997 | Still birth | 1.032 | 0.819 | 1.245 | -0.048 |
| Smith et al.1997 | Still birth | 1.092 | 0.911 | 1.273 | 0.012 |
| Varma and Patel 1987 | Still birth | 1.080 | 0.900 | 1.260 | 0.000 |
| Yu et al. 2018b | Still birth | 1.082 | 0.902 | 1.262 | 0.002 |
| Allen et al. 2022 (singletons) | Very high birthweight | 1.153 | 0.886 | 1.421 | 0.004 |
| Allen et al. 2022 (twins) | Very high birthweight | 1.147 | 0.999 | 1.296 | -0.002 |
| Kamath et al. 2018 | Very high birthweight | 1.150 | 0.974 | 1.325 | 0.001 |
| Adams et al. 2017 | Very low birthweight | 0.870 | 0.786 | 0.954 | -0.002 |
| Allen et al. 2022 (singletons) | Very low birthweight | 0.851 | 0.755 | 0.947 | -0.021 |
| Allen et al. 2022 (twins) | Very low birthweight | 0.911 | 0.784 | 1.038 | 0.039 |
| Gao et al. 2022 | Very low birthweight | 0.877 | 0.792 | 0.961 | 0.004 |
| Kamath et al. 2018 | Very low birthweight | 0.859 | 0.769 | 0.950 | -0.013 |
| Liu et al. 2017 | Very low birthweight | 0.878 | 0.794 | 0.963 | 0.006 |
| Varma and Patel 1987 | Very low birthweight | 0.874 | 0.788 | 0.961 | 0.002 |
| Yu et al. 2018 | Very low birthweight | 0.877 | 0.779 | 0.975 | 0.005 |
| Adams et al. 2017 | Very preterm birth | 0.867 | 0.787 | 0.947 | -0.002 |
| Allen et al. 2022 (singletons) | Very preterm birth | 0.895 | 0.801 | 0.989 | 0.026 |
| Allen et al. 2022 (twins) | Very preterm birth | 0.867 | 0.763 | 0.971 | -0.002 |
| Gao et al. 2022 | Very preterm birth | 0.872 | 0.793 | 0.952 | 0.004 |
| Kamath et al. 2018 | Very preterm birth | 0.870 | 0.784 | 0.955 | 0.001 |
| Varma and Patel 1987 | Very preterm birth | 0.869 | 0.790 | 0.949 | 0.000 |
| Yu et al. 2018 | Very preterm birth | 0.850 | 0.764 | 0.935 | -0.019 |
| Malchau et al. 2014 | Very preterm birth | 0.864 | 0.784 | 0.944 | -0.005 |

**Figure S1**

**Figure S2**

**Figure S3**

**Figure S4**


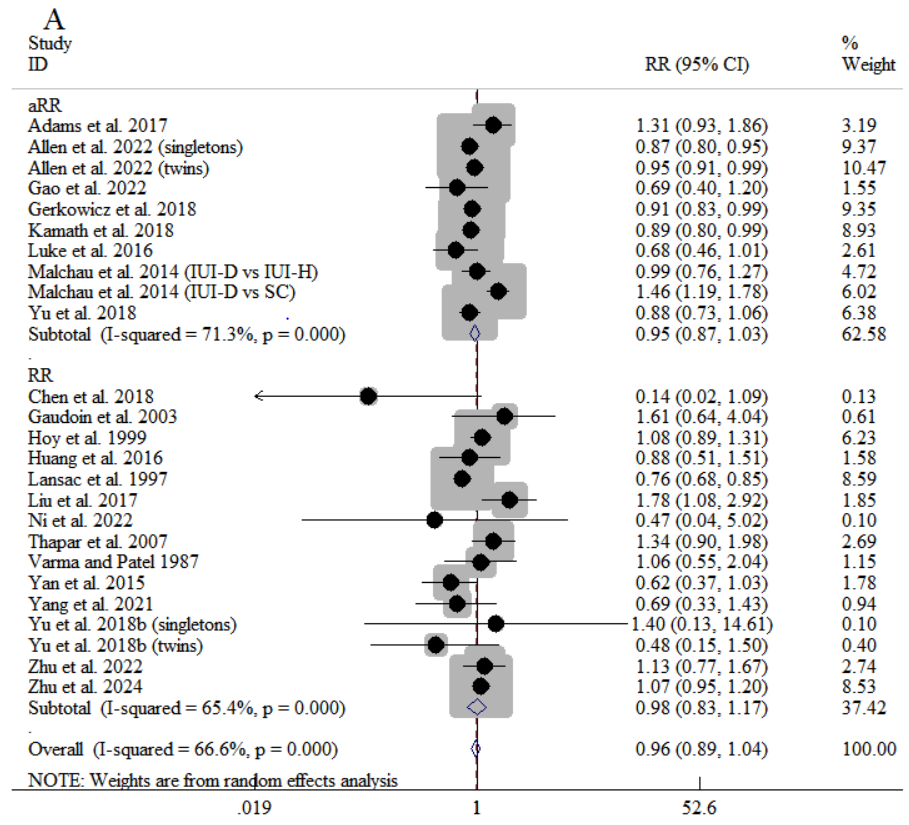

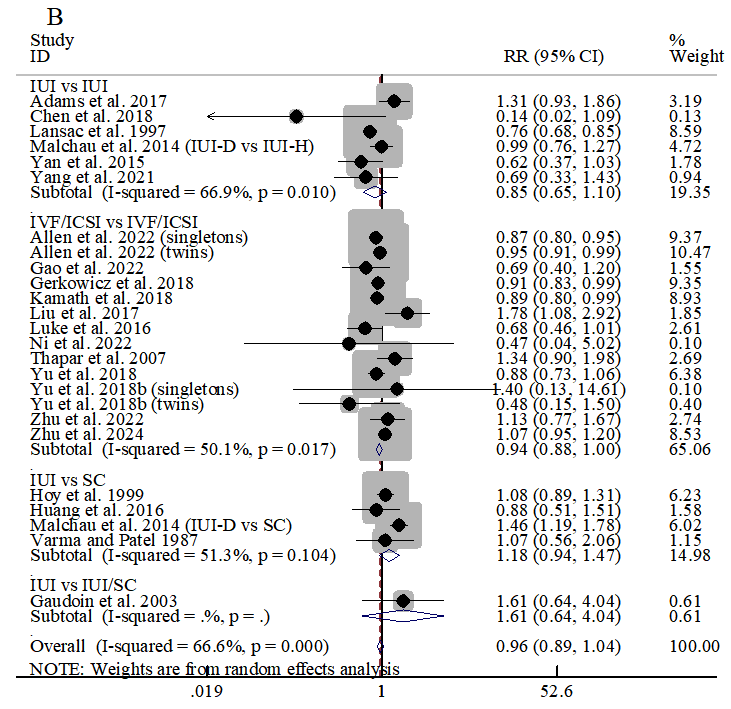

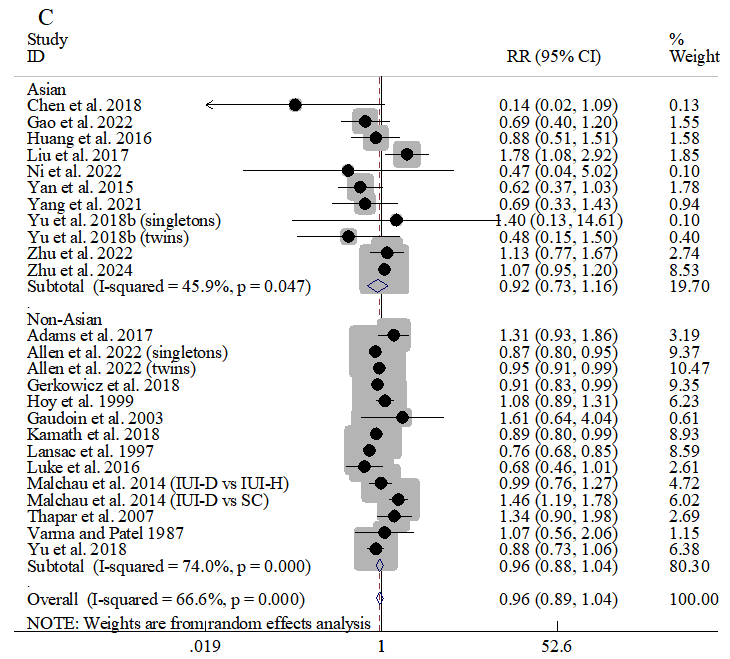


**Figure S5**

**Figure S6**

**
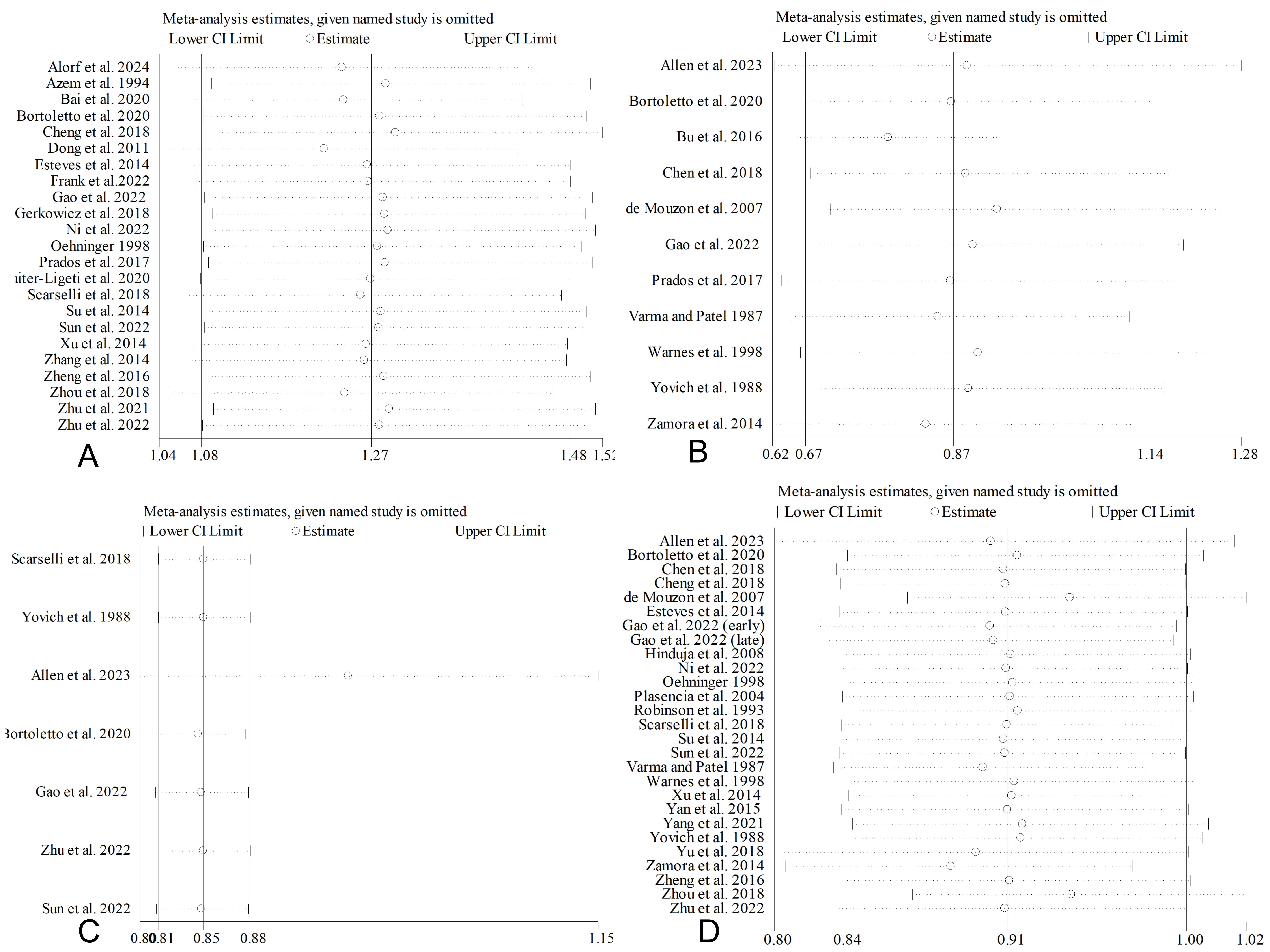
**

**Figure S7**

**
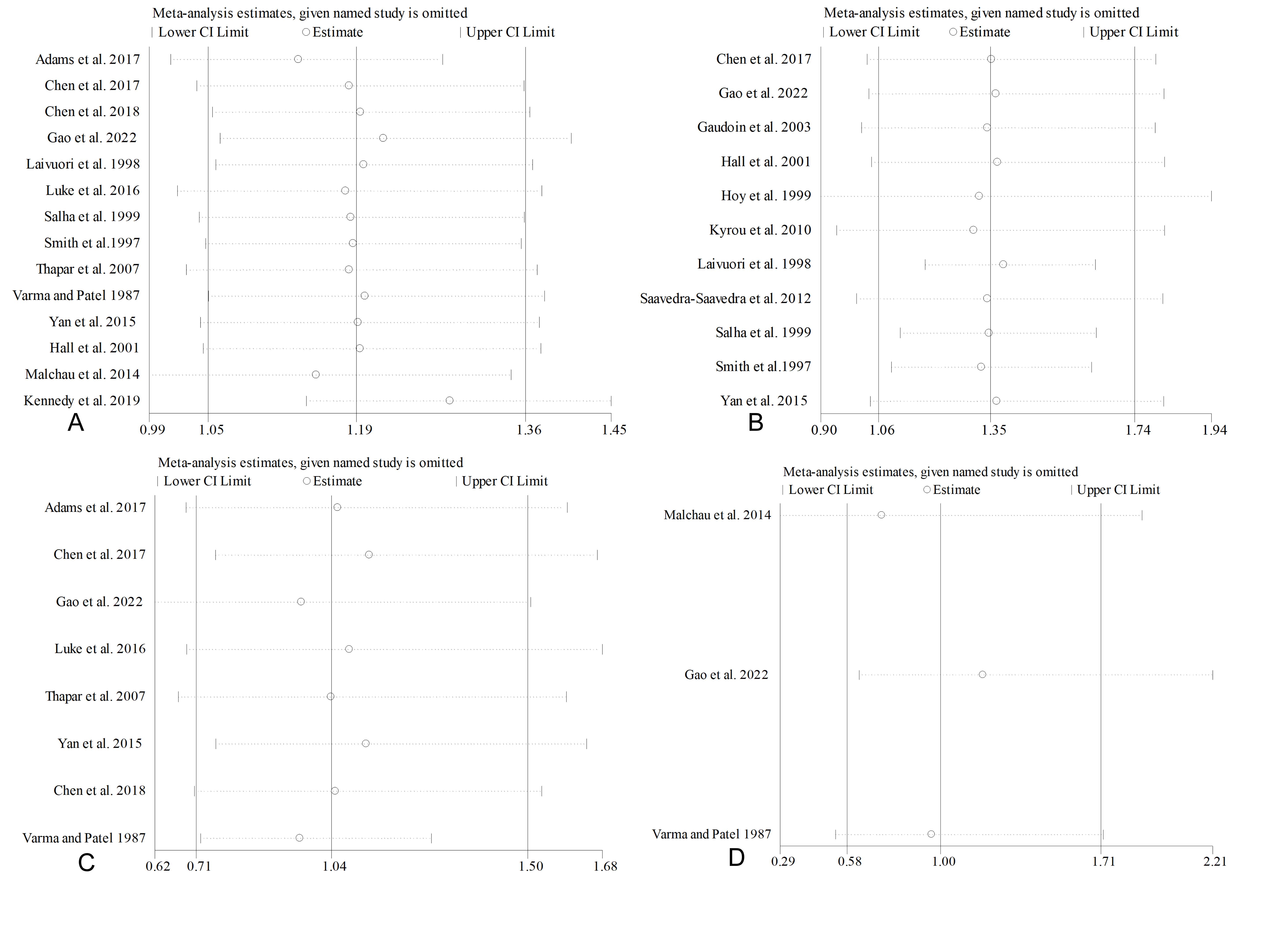
**

**Figure S8**

**
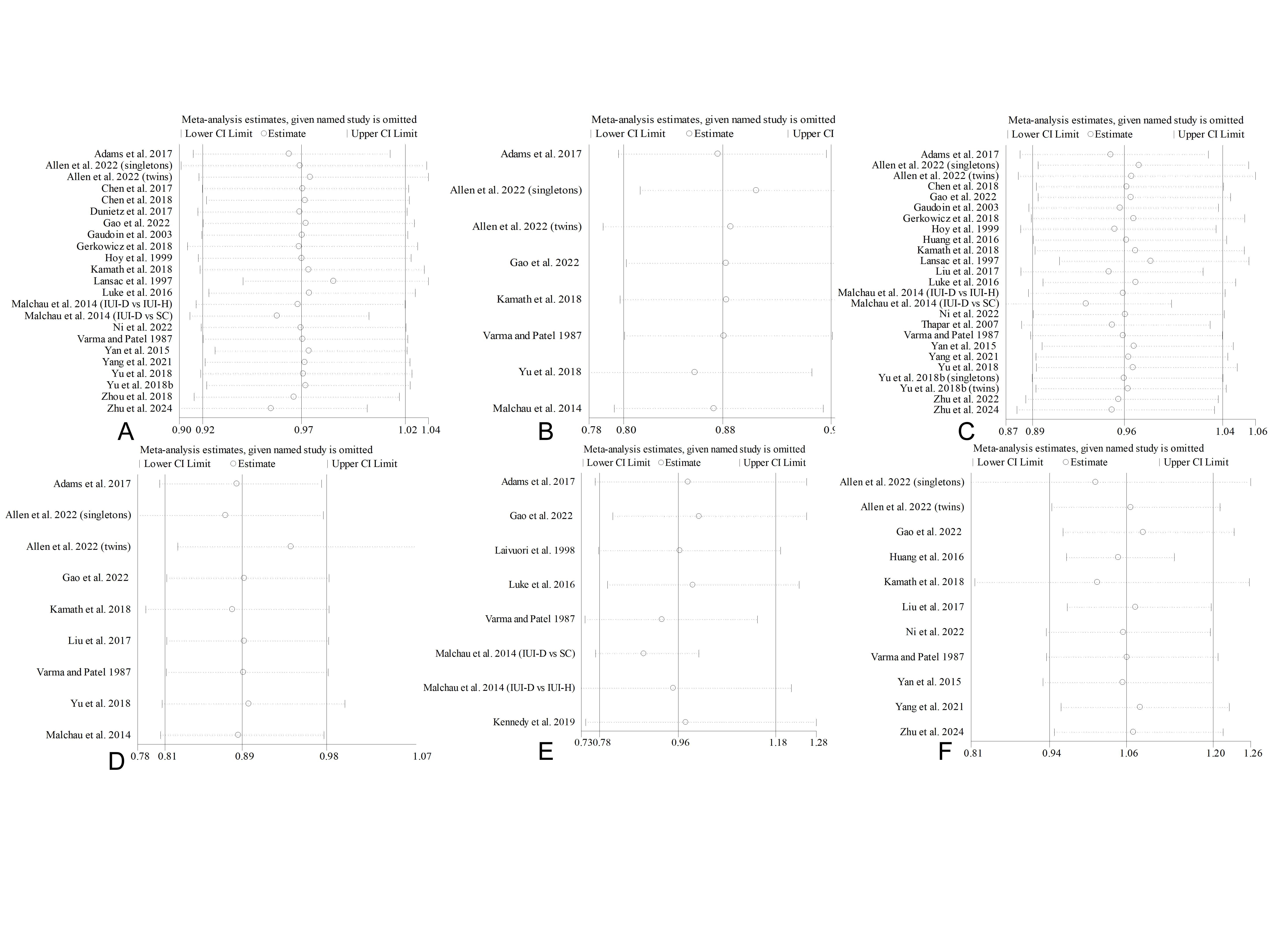
**

**Figure S9a**

**
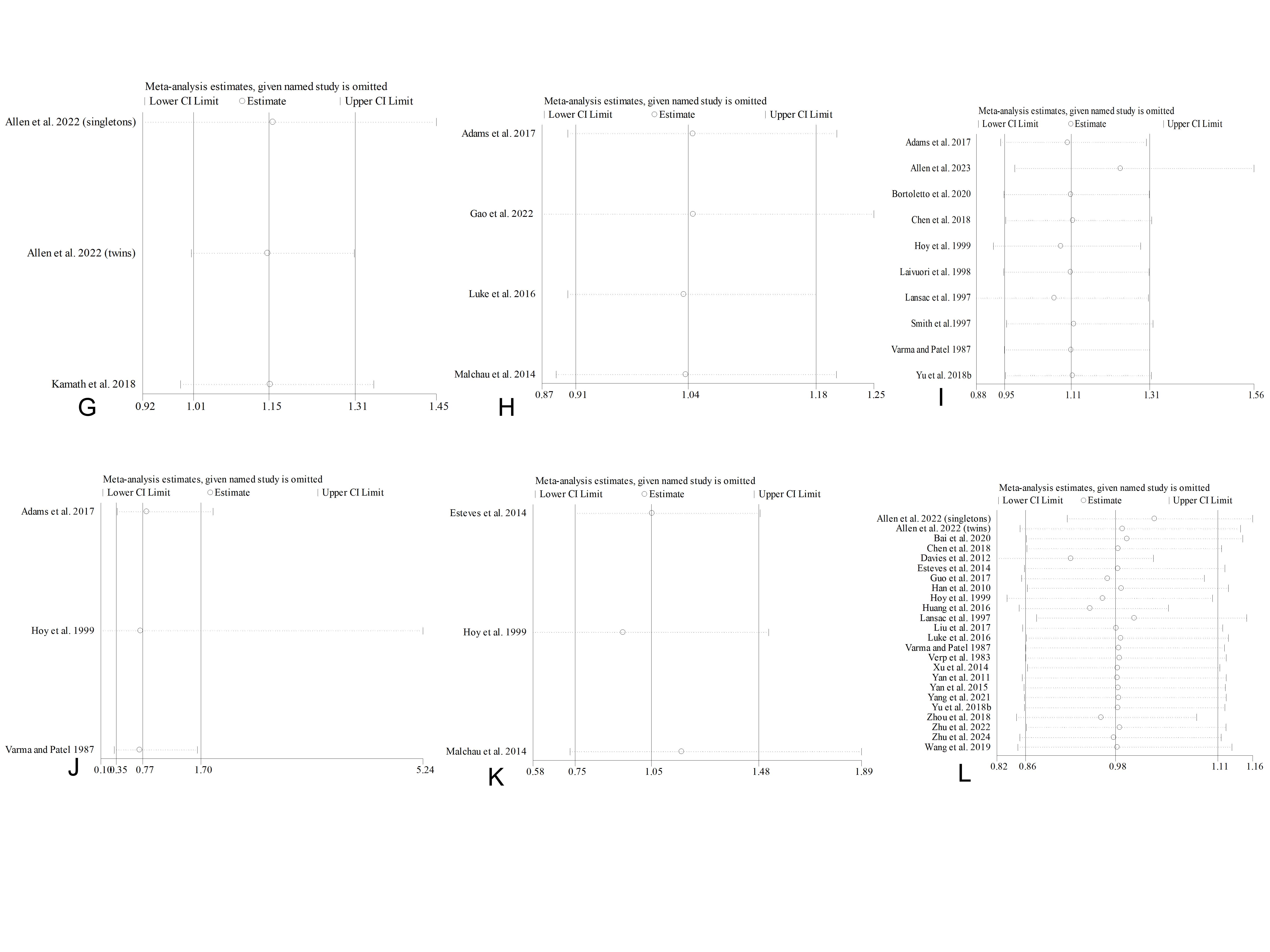
**

**Figure S9b**

**
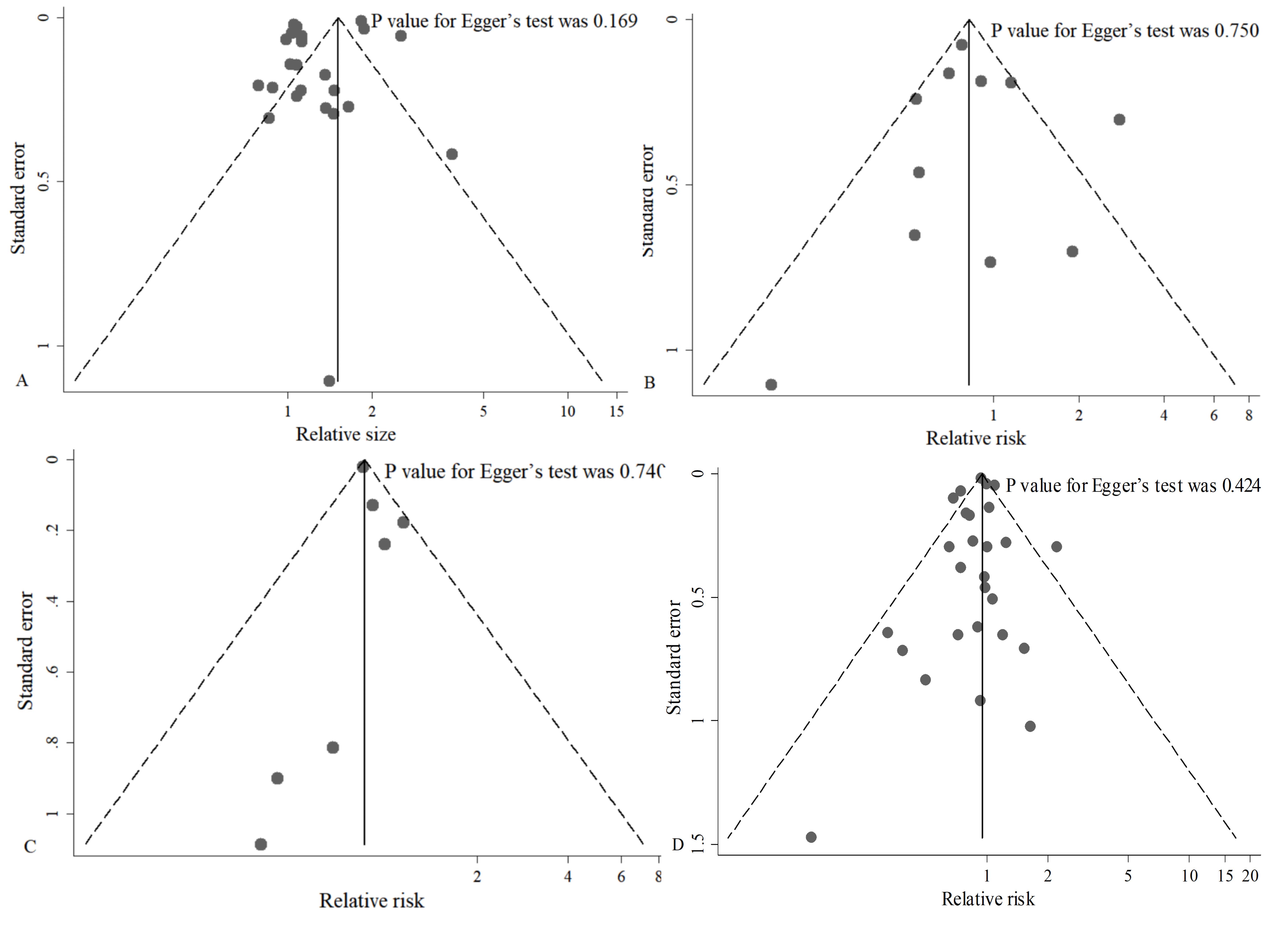
**

**Figure S10**

**
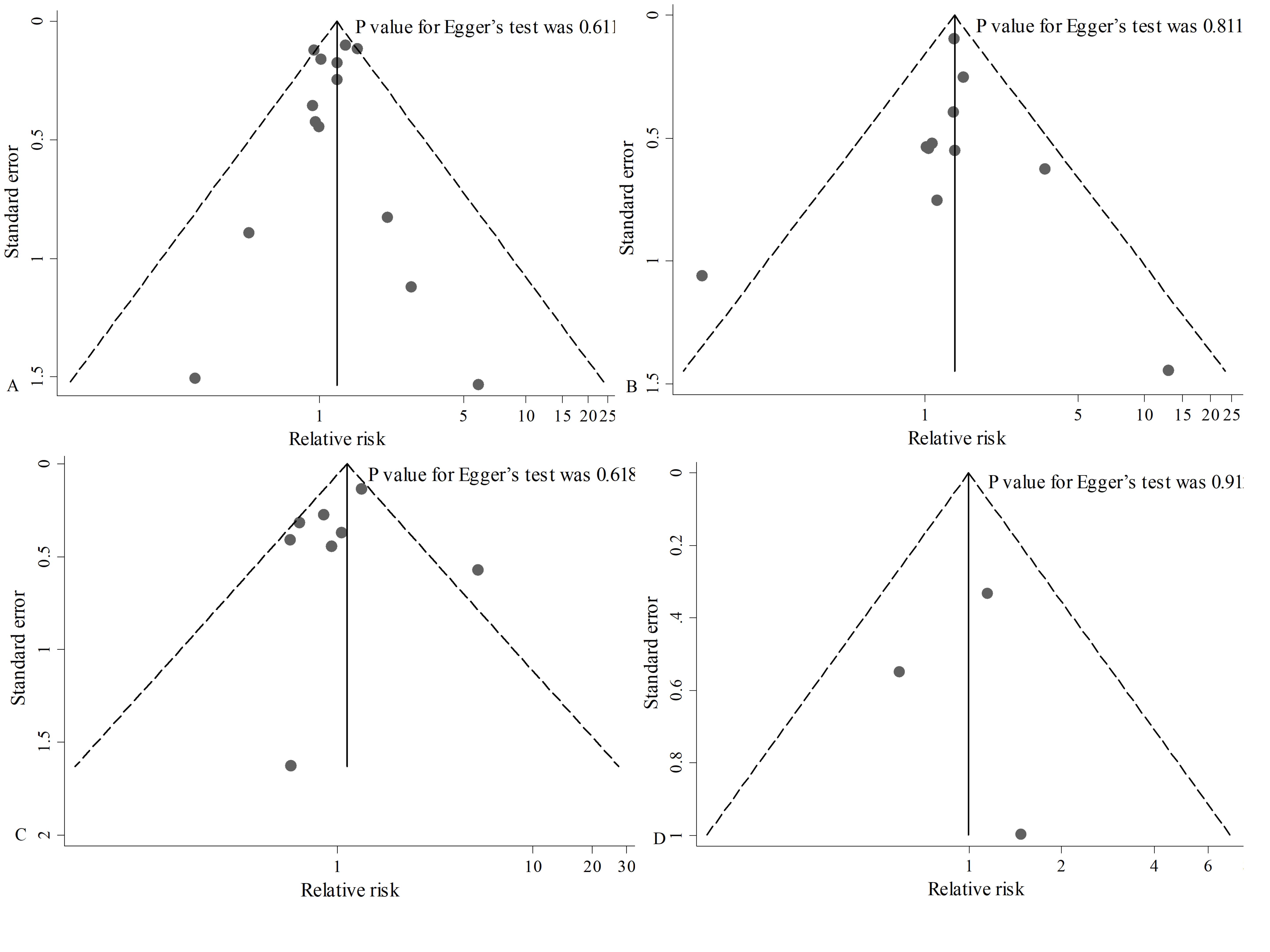
**

**Figure S11**

**
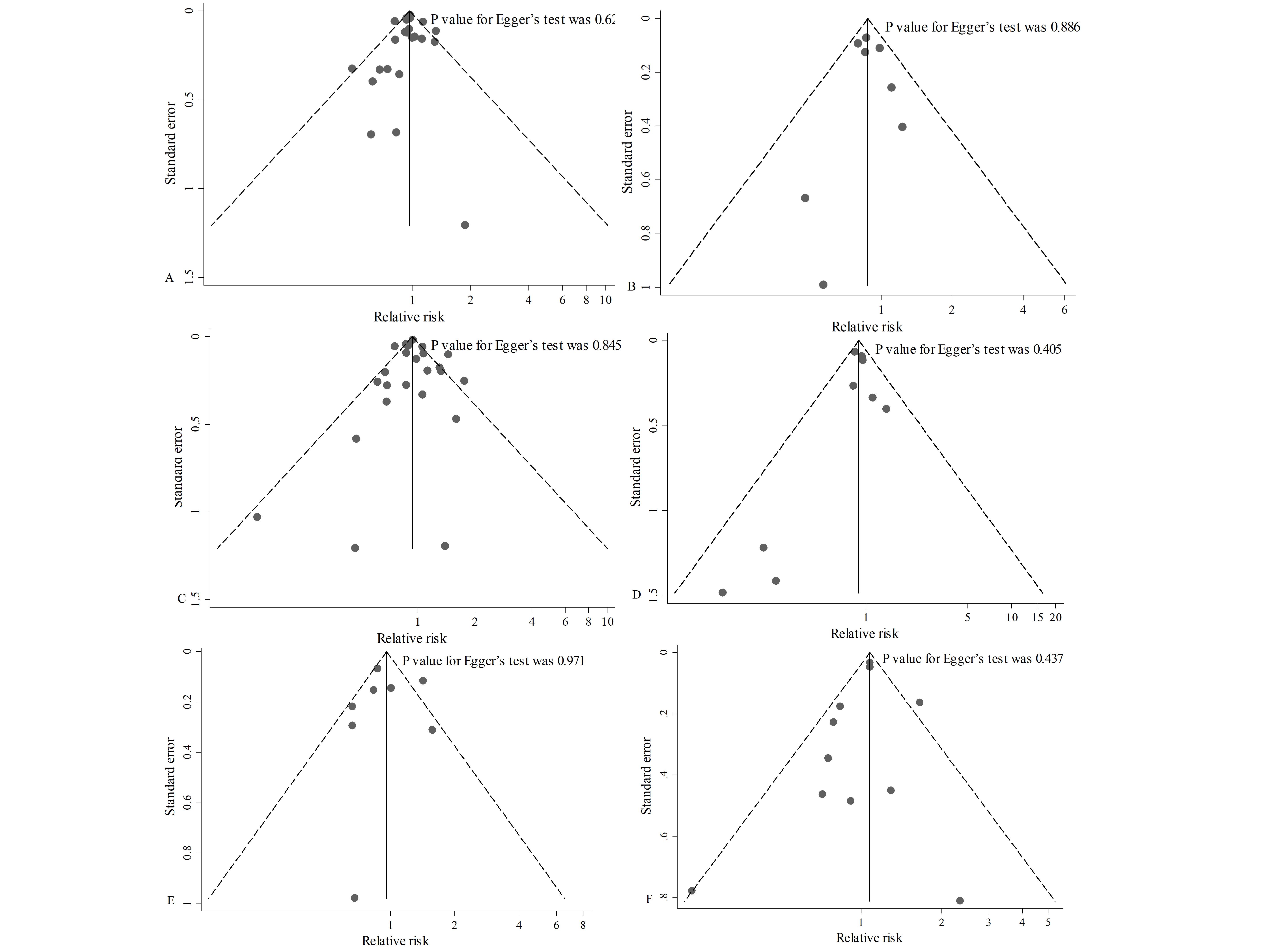
**

**Figure S12a**

**
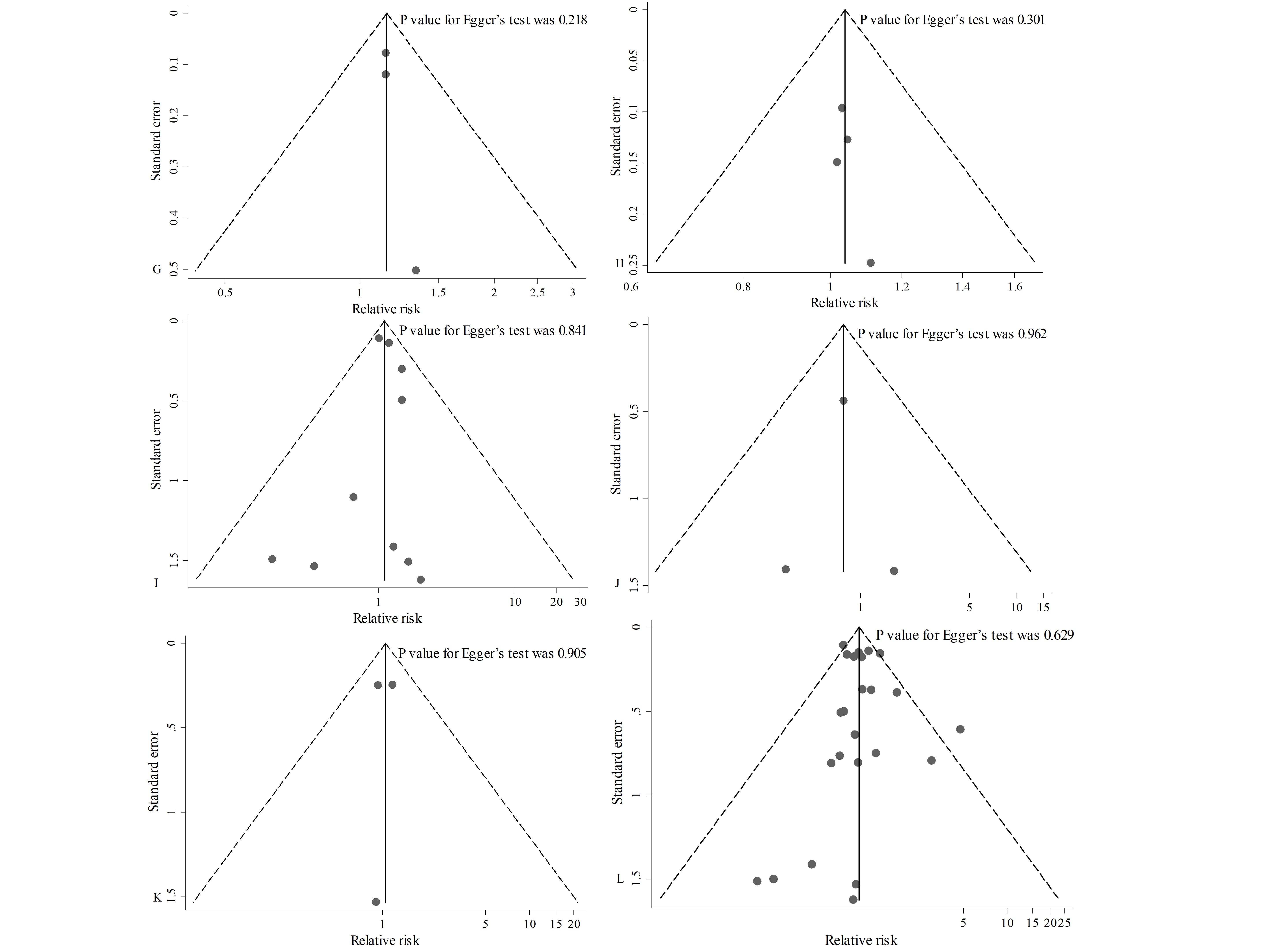
**

**Figure S12b**
